# Giant organelle vesicles to uncover intracellular membrane mechanics and plasticity

**DOI:** 10.1038/s41467-024-48086-7

**Published:** 2024-05-04

**Authors:** Alexandre Santinho, Maxime Carpentier, Julio Lopes Sampaio, Mohyeddine Omrane, Abdou Rachid Thiam

**Affiliations:** 1 Laboratoire de Physique de l’École normale supérieure, ENS, Université PSL, CNRS, Sorbonne Université, Université Paris Cité, F-75005 Paris, France; 2 Institut Curie, PSL Research University, Plateforme de Métabolomique et Lipidomique, 26 rue d’Ulm, Paris, France

**Keywords:** Membrane biophysics, Biophysical methods, Organelles

## Abstract

Tools for accessing and studying organelles remain underdeveloped. Here, we present a method by which giant organelle vesicles (GOVs) are generated by submitting cells to a hypotonic medium followed by plasma membrane breakage. By this means, GOVs ranging from 3 to over 10 µm become available for micromanipulation. GOVs are made from organelles such as the endoplasmic reticulum, endosomes, lysosomes and mitochondria, or in contact with one another such as giant mitochondria-associated ER membrane vesicles. We measure the mechanical properties of each organelle-derived GOV and find that they have distinct properties. In GOVs procured from Cos7 cells, for example, bending rigidities tend to increase from the endoplasmic reticulum to the plasma membrane. We also found that the mechanical properties of giant endoplasmic reticulum vesicles (GERVs) vary depending on their interactions with other organelles or the metabolic state of the cell. Lastly, we demonstrate GERVs’ biochemical activity through their capacity to synthesize triglycerides and assemble lipid droplets. These findings underscore the potential of GOVs as valuable tools for studying the biophysics and biology of organelles.

## Abstract (web summary)

The biophysical properties of cellular organelles are difficult to study directly. Here, the authors generate and characterize osmotically-expanded giant vesicles of several organelles, which maintain some of their functional properties.

## Introduction

The capacity to reconstitute elementary reactions remains crucial for the in-depth understanding of biology processes^1^. Such an asset has been made possible by major advances in biochemistry, bottom-up cell biology, synthetic biology, and biophysics. Despite the progress, manipulating intracellular organelles has remained challenging due to cell complexity, the small dimensions of organelles, and the physical barrier that represents the plasma membrane. Reconstituting or accessing manipulable organelles would allow studying better their structures and functions^2–4^.

Giant unilamellar vesicles (GUVs) generation offers versatile biomimetic membranes. They can be made out of synthetic or purified lipids and can be tens of micrometers in size. While they enable to study diverse biophysical and biochemical mechanisms^5^, they do not fully capture the complexity of cell membranes. Accurately controlling the lipid composition and leaflet distribution to match that of organelles remains challenging with GUVs. Furthermore, the incorporation of functional transmembrane proteins is non-trivial^6^.

A range of techniques allows for studying organelles such as their organization and dynamics. High-resolution imaging techniques^3,7,8^, such as expansion microscopy which expands nanometric dimensions to millimeters^9,10^, and advanced fluorescence microscopy methods, offer a good appreciation of organelle structures and dynamics, in intact cell conditions. Yet, such approaches do not or difficulty allow for direct manipulation of membranes. On the other hand, subcellular fractionation assays enable the isolation of functional organelles^11^, reproducing and characterizing enzymatic activities. However, the nanometric size of the recovered fragments represents a limitation for biophysical manipulations. In contrast, the recovery of giant plasma membrane vesicles (GPMVs) from cells undergoing blebbing or vesiculation has been useful for characterizing the biophysical cell plasma membrane properties by micropipette techniques^12–15^. By the same token, generating giant vesicles from intracellular organelles could improve our understanding of intracellular organelles’ biology. But, compared to the generation of GPMVs, which is relatively straightforward due to exposed Plasma membranes, creating giant vesicles from intracellular mammalian organelles remains less obvious. Recently, endoplasmic reticulum (ER) membrane-derived vesicles from Saccharomyces cerevisiae were reconstituted by the fusion of microsomal fragments’ after purification and subsequent GTP supply^16^. While promising, this approach involves several steps that may be limiting for routine usage in mammalian cells.

Here, we propose a method to generate functional giant vesicles deriving from intracellular bilayer-bounded organelles. We investigated their biophysical properties depending on their nature, location, and cell metabolic state. We demonstrated that vesicles arising from the ER are biochemically functional based on their capacity to synthesize neutral lipids and support de novo organelle biogenesis.

## Results and discussion

### Generation of Giant Organelle Vesicles (GOVs)

**Fig. 1:**
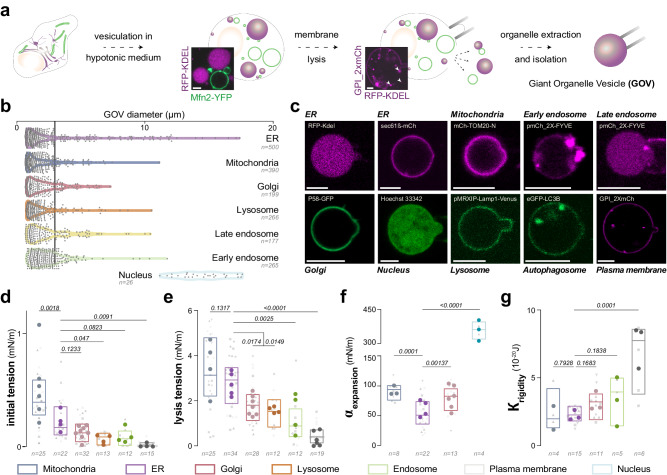
Giant Organelles Vesicles’ recovery and characterization. **a** Organelles reshaping into Giant Organelle Vesicles (GOVs) by a hypotonic medium. Confocal microscopy image of a swollen COS-7 cell expressing RFP-KDEL and Mfn2-YFP. Confocal microscopy snapshot showing the release of Giant ER Vesicles (GERVs) after plasma membrane rupture with a micropipette. Cells expressing GPI_2x-mCherry (Plasma Membrane) and RFP-KDEL(ER). Scale bar: 5 µm. Schematic representation of a collected GOV. **b** Diameter distribution of Giant Organelle Vesicles (GOV). Only vesicles larger than 0.75 µm were measured. For each organelle type, quantifications were done from two independent experiments with hundreds of vesicles quantified from tens of cells. **c** Confocal images of collected Giant Organelle Vesicles (GOVs) labeled with fluorescence markers representing different organelles. Scale bars: 5 µm. **d** Plot illustrating the initial membrane tension of Giant Organelle Vesicles (GOVs). For organelles from left to right, the number of independent experiments is *N* = 6; *N* = 4; *N* = 8; *N* = 4; *N* = 4; *N* = 4. Each experimental data point corresponds to a GOV from a different cell and the mean of each replicate is shown. Nested One-Way ANOVA - Multiple comparisons: Fisher LSD test. Individual *P* values are shown. **e** The lysis tension of Giant Organelle Vesicles (GOVs). For organelles from left to right, the number of independent experiments is *N* = 4; *N* = 4; *N* = 7; *N* = 4; *N* = 4; *N* = 4. Each experimental data point corresponds to a GOV from a different cell and the mean for each replicate is shown. Nested One-Way ANOVA - Multiple comparisons: Fisher LSD test. Individual P values are shown. **f** The apparent area expansion modulus of Giant Organelle Vesicles (GOVs). For organelles from left to right, the number of independent experiments is *N* = 4; *N* = 6; *N* = 3; *N* = 3. Each experimental data point corresponds to a GOV from a different cell and the mean for each replicate is shown. Nested One-Way ANOVA - Multiple comparisons: Fisher LSD test. Individual P values are shown. **g** Bending rigidity of Giant Organelle Vesicles (GOVs). For organelles from left to right, the number of independent experiments is *N* = 2; *N* = 3; *N* = 3; *N* = 3, *N* = 3. Each experimental data point corresponds to a GOV from a different cell and the mean for each replicate is shown. Nested One-Way ANOVA - Multiple comparison: Fisher LSD test. Individual *P* values are shown.

We subjected Cos7 cells to a hypotonic solution, which swelled the cells, made them spherical and tenser, and so did their intracellular bilayer-encircled organelles (Fig. 1a, supplementary Fig. 1a–g). This process led to the formation of giant organelle vesicles (GOVs)^17–19^ having sizes that can be larger than 3 µm (Fig. 1b, supplementary Fig. 1h), except for peroxisomes^18^; giant Golgi vesicles were less frequent than other organelles. To identify GOVs, different fluorescent protein markers were transfected to the cells (Fig. 1c, supplementary Fig. 1b–g): KDEL or Sec61 for ER, Tom20, Mito-BFP or Mitofusin for Mitochondria, FYVE for early and late Endosomes, Golgi-7 or P58 for Golgi, Lamp1 for Lysosome, LC3B for autophagosome and autolysosome, GPI for the Plasma membrane. With our GOV recovery protocol, we also observed the formation of giant plasma vesicles, which might be distinct from GPMVs generated by cell blebbing induced by chemical vesiculants^20^.

The size of giant ER vesicles (GERVs), up to 20 µm, was on average larger than that of other organelles, except for the nucleus (Fig. 1b, supplementary Fig. 1h). Since the swelling process should minimize the surface-to-volume ratio of the organelles, we hypothesized that the size of GOVs should be dictated by the endogenous surface-to-volume ratio of their corresponding organelles, i.e., their aspect ratio. To test this, we overexpressed Climp-63, promoting ER sheets over tubules^21^, and, therefore, increasing the endogenous surface-to-volume. Under this condition, the swelling of the cells led to the formation of much larger ER vesicles, sometimes, almost occupying the entire cytosol (supplementary Fig. 1i, k–m). Likewise, nocodazole treatment, which disrupts microtubules and decreases ER tubules, increased GERVs’ average size (supplementary Fig. 1j, k–m). To further test our hypothesis, we overexpressed Mitofusin-2, which promotes mitochondrial elongation by fusion. Likewise, we found that the average size of giant mitochondrial vesicles (GMV) increased compared to GMVs from non-overexpressed conditions (Supplementary Fig. 1n–p). These experiments on GERVs and GMVs suggest that the sizes of GOVs are determined by the organelles’ native aspect ratio.

We micromanipulated the swollen cells to lyse them by increasing their plasma membrane tension (Fig. 1a). Upon cell breakage, GOVs were released to the extracellular medium (Fig. 1c). This suction strategy allowed us to break one cell at a time. Alternatively, with a pipette, we applied aspiration and refill cycles to the medium containing the cells, and this led to the lysis of many cells. This approach was effective on many cell lines including Cos7, HeLa, Huh7, and fibroblasts (supplementary Fig. 2a–f), validating the versatility of the approach. Hereafter, we only worked on GOVs obtained from Cos7 cells.

### GOVs from different organelles have distinct mechanical properties

We isolated independent GOVs and measured their biophysical properties. For different organelles, we determined, when it was possible, the initial surface tension, lysis tension, apparent area expansion modulus or stretching rigidity (${\alpha }_{{expansion}}$), and bending rigidity (K) (Fig. 1d–g, Supplementary Fig. 2g–u).

The initial tension of giant Plasma membrane vesicles made during our GOVs’ preparation was 0.02 ± 0.01mN/m (Fig. 1d), which is close to values reported for GPMVs or cells subjected to hypotonic media^7,15^. The plasma membrane was followed by early endosomes (GEVs, 0.07 ± 0.05mN/m) and lysosomes (GLVs, 0.08 ± 0.03mN/m) (Fig. 1d). Mitochondria had the highest value (GMVs, 0.44 ± 0.11mN/m), then the ER (GERVs, 0.24 ± 0.08mN/m) and the Golgi (GGVs, 0.14 ± 0.04mN/m) (Fig. 1d). These tension values are not the native values because of the induced organelles swelling.

Next, we measured the GOV’s lysis tension (Fig. 1e), reflecting how much the membrane sustains stretching at a given rate, which was identically for all GOV types. Lysis tensions were between 1.1 ± 0.7mN/m and 3.3 ± 0.7mN/m (Fig. 1e). Plasma membrane vesicles, which formed during the GOVs’ extraction, had the lowest lysis tension (0.5 ± 0.2mN/m), followed by GEVs (1.1 ± 0.7mN/m), GLVs (1.6 ± 0.5mN/m), GGVs (1.9 ± 0.3mN/m), GERVs (2.8 ± 0.4mN/m), and GMVs (3.3 ± 0.7mN/m). These measurements indicate that the lysis tension gradually decreases from the ER/Mito to the Plasma membrane, i.e. along the secretory pathway. Interestingly, even though the initial tensions do not mirror those of the intact organelles, they also displayed a similar decrease along the organelles of the secretory pathway. This trend aligns with measurements conducted on intact organelles utilizing fluorescent tension probes or optical tweezers^7,22^.

It is worth noting that the measured GOVs’ lysis tensions are much lower than for GUVs (around 8-10mN/m)^23^, reflecting the gap between synthetic and organellar membranes. Such discrepancies likely stem from the complex lipid mixture and organization in cell membranes as compared with GUVs, in addition to the presence of diverse proteins impacting membrane properties.

Next, we measured the apparent area expansion modulus, which is an inherent membrane property. The nucleus had the highest modulus (356 ± 61mN/m), followed by GMVs ( ~ 92 ± 13mN/m), GGVs ( ~ 78 ± 15mN/m), and GERVs ( ~ 55 ± 12mN/m) (Fig. 1f). For GMVs and the Nucleus in particular, we cannot exclude possible lipid exchanges between the inner and outer area which would impact the expansion modulus. The modulus of the ER was lower than the one of the Golgi, whose value was lower than that of GPMVs^15,24^. Again, on average, these area expansion moduli are discrepant from that of dioleoyl phosphatidylcholine GUVs ( ~ 200mN/m)^23,25^.

Regarding the bending rigidity, GERVs and GMVs had similar and the lowest rigidities ( ~ 6 k_B_T) (Fig. 1g). They were followed by GEVs and GGVs ( ~ 8 k_B_T). These values were lower than that of plasma membrane vesicles generated during GOVs’ recovery (17 k_B_T) (Fig. 1g), which is close to the rigidity of GPMVs and GUVs^15^. These findings argue that membrane rigidities increase from the ER to the Plasma membrane, opposite to the trend of tensions^7,22^, and partially mirror the expansion modulus evolution across organelles. These gradients could potentially be attuned to the gradients along the secretory pathway in lipids, membrane thickness, sterol content, and acyl chain saturation^26,27^, which, collectively, influence membrane mechanical properties^23^. The existence of these mechanical gradients suggests a potential mechanism for encoding how mechanical information propagates from the plasma membrane to the cell interior^28,29^.

Despite our above findings, interpretations of our GOVs’ results warranted some caution. First, during our vesiculation process of organelles by the hypotonic medium, many tiny vesicles also form (Fig. 1b) and could have been different in composition than GOVs^17^, meaning that the latter may partially recapitulate organelles’ properties. Secondly, we asked about the impact of protein overexpression. We took GERVs made from cells overexpressing Sec61ß or Sec22, which are integral ER membrane proteins with no known related functions (translocon and a SNARE respectively), or KDEL, which is an ER luminal signal peptide. We found that such GERVs had similar properties between Sec61 and KDEL (supplementary Fig. 2v), and slight differences appeared between Sec22 and Sec61. This disparity could be due to the possibility that Sec22 and Sec61 delineate distinct ER regions or their differing functions. We cannot exclude that the overexpression of certain other proteins might display more discrepancies. This is the reason why we privileged the overexpression of luminal proteins whenever possible, e.g. KDEL for GERVs, to minimize alteration in the organelle membrane properties. Thirdly, organelles within intact cells exhibit dynamic heterogeneity, making it challenging to comprehensively measure their properties due to constant fluxes and non-equilibrated conditions. The creation of GOVs might capture only a “frozen” state, offering a snapshot of organelle properties at a specific moment. Nevertheless, this enables the observation and quantification of variations and heterogeneities in organelle properties.

### GERV mechanical properties vary across contact sites

**Fig. 2:**
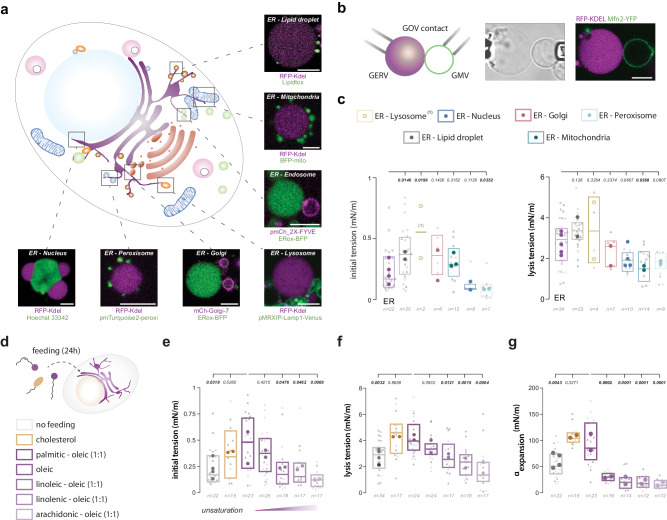
Biophysical properties of the ER at its contact regions and under different metabolic conditions. **a** Diagram representing a cell with highlighted contacts between the ER and other organelles. Each contact is depicted with a confocal microscopy snapshot showing a Giant ER Vesicle (GERV) in contact with other GOVs. Fluorescent proteins used to visualize the contacts are indicated below each snapshot. **b** Left: Diagram of an isolated contact between a Giant ER Vesicle (GERV) and a Giant Mitochondrial Vesicle (GMV). Right: Confocal microscopy snapshot of a KDEL-based GERV in contact with an Mfn2-based GMV after isolation. The GOVs were immobilized using micropipettes for imaging. Scale bar: 5 µm. **c** Left: Initial membrane tensions of KDEL-based Giant ER Vesicles (GERVs) in contact with other Giant Organelle Vesicles (GOVs). From left to right, the number of independent experiments is *N* = 2; *N* = 2; *N* = 3; *N* = 3, *N* = 2; *N* = 2; *N* = 4. Right: Lysis tensions of KDEL-based GERVs in contact with other GOVs. From left to right, the number of independent experiments is *N* = 2; *N* = 2; *N* = 2; *N* = 2, *N* = 4; *N* = 2; *N* = 4. Only RFP-KDEL and ERox-BFP luminal markers were used to identify the ER. Nested One-Way ANOVA - Multiple comparisons: Fisher LSD test. Individual *P* values are shown. **d** Feeding conditions. Following transfection, the cells were loaded with a combination of fatty acids or cholesterol. **e** Initial membrane tensions of Giant ER Vesicles (GERVs) after 24 hours of feeding. From left to right, the number of independent experiments is *N* = 4; *N* = 2; N = 2; *N* = 2, *N* = 2; *N* = 2; *N* = 2. Each experimental data point corresponds to a Giant Organelle Vesicle (GOV) from a different cell and the mean for each replicate is shown. Nested One-Way ANOVA - Multiple comparisons: Fisher LSD test. Individual P-values are shown. **f** Lysis tensions of Giant ER Vesicles (GERVs) after 24 hours of feeding. From left to right, the number of independent experiments is *N* = 4; *N* = 2; *N* = 2; *N* = 2, *N* = 2; *N* = 2; *N* = 2. Each experimental data point corresponds to a GOV from a different cell and the mean for each replicate is shown. Nested One-Way ANOVA - Multiple comparisons: Fisher LSD test. Individual *P* values are shown. Refer to the Statistical Tests section or Source Data for statistical analysis and data sets. **g** Apparent area expansion modulus of Giant ER Vesicles (GERVs). From left to right, the number of independent experiments is *N* = 4; N = 2; *N* = 2; *N* = 2, *N* = 2; *N* = 2; *N* = 2. Each experimental data point corresponds to a GOV from a different cell and the mean for each replicate is shown. Nested One-Way ANOVA - Multiple comparisons: Fisher LSD test. Individual P-values are shown. Refer to the Statistical Tests section or Source Data for statistical analysis and data sets.

In the above experiments, we worked with individual GOVs but different GOV types were also in contact (Fig. 2a, Supplementary Fig. 3a–c). GERVs in particular were central to these contacts, which may not be surprising given the ER membrane is the largest organelle that organizes most cellular functions^2,3^ (Supplementary Fig. 3d–n). We measured the surface tension of GERVs recovered in contact with different GOV types (Fig. 2a, b). Because of the contacts, measuring the other biophysical parameters was difficult due to possible crosstalk with the contacting GOVs.

We selected KDEL- or ERox-based GERVs that were primarily in contact with one different GOV type (Fig. 2b, supplementary Fig. 3d–n). GERVs in contact with GLVs had the highest initial bilayer tension (0.55 mN/m), followed by those in contact with GGVs or lipid droplets (0.37mN/m) and GMV (0.31mN/m) (Fig. 2c). These tensions were on average larger than that of individual GERVs (0.24mN/m) (Fig. 1). In contrast, GERVs in contact with peroxisomes and the nucleus, i.e., the nuclear ER, had lower tensions (0.13mN/m). Regarding the lysis tension, it was slightly higher for GERVs in contact with GLVs and lipid droplets (3.5mN/m) than for single GERVs (2.7mN/m) (Fig. 2c). GERV-Golgi (Golgi7) had similar lysis tension than individual GERVs when GERVs in contact with the nucleus, peroxisomes, and GMVs had lower lysis tensions ( ~ 2mN/m).

The distinct tension values at GERV contact sites may reflect or be reflected by the heterogeneity in the ER, in shape, thickness, composition, and function^30–34^. Threefold differences could exist between the nuclear-ER and the ER region in contact with lysosomes (Fig. 2c). These findings raise questions about the potential presence of surface tension gradients along the ER. As the ER is a continuous network, such gradients could induce transient membrane flows, directing proteins and lipids to specific ER regions.

### The influence of lipid uptake on the physical properties of GERVs

Lipid saturation or cholesterol is among the key membrane factors determining the integrity, fluidity, and overall functionality of membranes^35–39^. We explored the extent to which the supply to cells of these lipids alters the properties of the ER.

To investigate the incorporation of fatty acids into ER membrane lipids, we conducted an experiment where cells were fed with different fatty acid mixtures for 24 hrs. Notably, two fatty acid mixtures were used: palmitic:oleic (1:1) and linoleic:linolenic (1:1), which contrast in saturation. After lysis and ultracentrifugation, microsomes were recovered and subjected to lipidomic analysis using mass spectrometry. The fed fatty acids were effectively integrated into ER membrane phospholipids, showcasing a variety of acyl chain lengths and compositions. The protein levels in the ER remained largely unchanged (supplementary Fig. 4a–j).

**Fig. 3:**
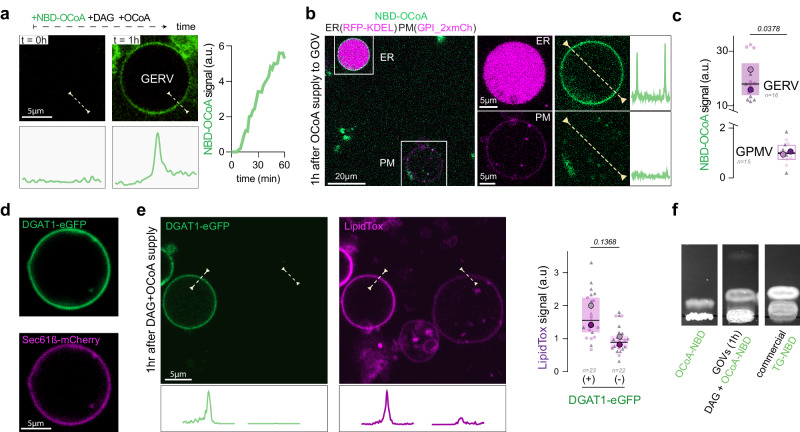
GERVs are functional and accommodate the synthesis of neutral lipids. **a** Left: Confocal microscopy of a Giant ER Vesicle (GERV) supplied with Oleoyl-CoenzymeA (OCoA) supplemented with NBD-Oleoyl-Coenzyme A (NBD-OCoA) and diacylglycerol (DAG) at 37 °C for 1 hour. Fluorescence signals at the bottom of the snapshots correspond to the profile indicated by the dotted line on the confocal images. Scale bar: 5 µm. Right: Plot showing the increase in NBD-OCoA fluorescence signal in the GERV membrane during the OCoA supply experiment. For both conditions, the number of independent experiments is *N* = 2, and the mean for each replicate is shown. A nested t-test was applied with a two-tailed P-value. P-values are shown. **b** Left: Confocal images of both KDEL-based Giant ER Vesicle (GERV) and GPI-based Giant Plasma Membrane Vesicle (GPMV) after 1 hour of OCoA supply. Fluorescence signals at the right corner correspond to the profile indicated by the dotted line on the confocal images. Scale bars: 5 µm. **c** Plot displaying the NBD-OCoA fluorescence signal in Giant ER Vesicles (GERVs) and Giant Plasma Membrane Vesicles (GPMVs) after 1 hour of feeding. For both conditions, the number of independent experiments is *N* = 2, and the mean for each replicate is shown. A nested t-test was applied with a two-tailed P-value. P-values are shown. Refer to Statistical Tests section or Source Data for statistical analysis and data sets. **d** Giant ER Vesicles were isolated from COS-7 cells overexpressing the DGAT1-EGFP and Sec61ß-mCherry. **e** Left: Isolated Giant Organelle Vesicles were subjected to the feeding conditions as in a. LipidTox was added to reveal membrane hydrophobicity. Fluorescence signals at the bottom of the images correspond to the profile indicated by the dotted line on the confocal images. Scale bars: 5 µm. Right: Box plot showing the LipidTox fluorescence in vesicles positive (+) or negative (--) for DGAT1. The number of independent experiments is *N* = 2 and a nested t-test was applied (**f**). Thin layer chromatography snapshot revealed by UV light indicates TAG-NBD synthesis in GERVs after 1 hour of feeding. Left: Commercial sample of OCoA-NBA; middle: Lipid extract from GERVs fed with OCoA-NBD and DAG; right: Commercial sample of TAG-NBD.

Next, we fed cells for 24 hrs with fatty acids bearing different saturation levels, from the unsaturated palmitic acid to the polyunsaturated arachidonic acid, mixed with oleic acid (1:1), or cholesterol (Fig. 3d). For each condition, we generated GERVs and measured their physical properties. The impact of palmitic acid and cholesterol was consistently predominant as they corresponded to a noticeable increase in the lysis tensions ( ~ 4.25mN/m) and in the apparent area expansion modulus ( ~ 100mN/m), compared to control GERVs (Fig. 3e–g). In noticeable contrast, linoleic, linolenic, and arachidonic led to a gradual decrease in tension and a sharp decrease in the apparent area expansion modulus (15-25mN/m) (Fig. 3e–g). These parameters changed by almost 4 folds between palmitic and arachidonic acids, indicating the extent to which the ER mechanical properties may be altered by lipid saturation^36,37^; such mechanical alterations could be a proxy for ER stress or pathological conditions. For comparison, GUVs made with phospholipids bearing different saturation levels have apparent area expansion moduli, ranging from 150 to 300mN/m^23,25^, which is much larger than for GERVs.

Finally, we wondered whether the alteration in properties by fatty acid feeding could be reversed by starvation. A cell batch was unfed and placed in a DMEM for 48hrs. Another batch was fed with oleic acid in DMEM for 48hrs. A last sample was fed for 24h with oleic acid and then starved for 24h in EBSS for 24hrs. GERVs were generated from each condition and their membrane properties were measured. GERVs from the unfed and fed-starved conditions had similar properties (supplementary Fig. 4k), as if starvation after oleic acid feeding restored the membrane properties. In the fed condition, we observed slightly but significantly different values compared to the unfed and fed-starved conditions.

Together, our above measurements on GERVs reveal the level of changes in the properties of the ER membrane depending on metabolic cues.

### GERVs preserve ER functionalities

The ER is the biosynthesis site of lipids and proteins and the biogenesis site of several organelles^40^, notably lipid droplets (LDs). We tested whether GERVs can mimic biochemical and biophysical reactions necessary for LD biogenesis.

We made GERVs and supplied them with dioleoylglycerol (DAG), oleyl-CoA, containing 1% NBD-Oleoyl-CoA, which are substrates for triacylglycerol (TAG) synthesis. The fluorescent fatty acids enabled us to visualize the synthesis of neutral lipids by fluorescence^17^. We observed that GERVs’ membrane acquired the NBD-fluorescence over time (Fig. 3a, supplementary Fig. 5a, b). This increase in fluorescence was likely due to the synthesis of fluorescent TAG^17^, as bare artificial membranes with ER-like phospholipid composition within the same environment did not have the same NBD signal (supplementary Fig. 5c). Additionally, GPI_2x-labeled plasma membrane vesicles, which do not possess the TAG synthesis enzymes, almost lacked the NBD fluorescence (Fig. 3b, Supplementary Fig. 5d). To further evidence the synthesis of TAG, we overexpressed DGAT1-eGFP, responsible for converting DAG into TAG, and used LipidTox to reveal TAG production. We observed that DGAT1-eGFP-positive GERVs exhibited stronger LipidTox signals compared to other GOVs in their vicinity (Fig. 3d, e, Supplementary Fig. 5e, f). Finally, we used chromatography and mass spectrometry to evidence TAG synthesis. To do so, we first swelled cells and broke them by suction and refill of the cell-containing medium. The released GOVs were then incubated with the same cocktail for TAG synthesis. The sample was then recovered and analyzed by using thin-layer chromatography and lipidomics, which confirmed the incorporation of the provided NBD-Oleyl fatty acid into TAGs (Fig. 3f, Supplementary Fig. 5g, h). Collectively, these results support that GERVs preserved the catalytical activity of ER enzymes. Hence, we foresee other GOVs to be capable of reproducing biochemical reactions of the organelles they derive.

In our previous work using protein-free systems, we proposed that the condensation of TAG into LDs is catalyzed by membrane curvature^15^. In cells, this process is assisted by the integral ER membrane protein complex seipin^41–44^, which showed a preference for curved regions^17,45^. GERVs represent the best tool to test these hypotheses.

**Fig. 4:**
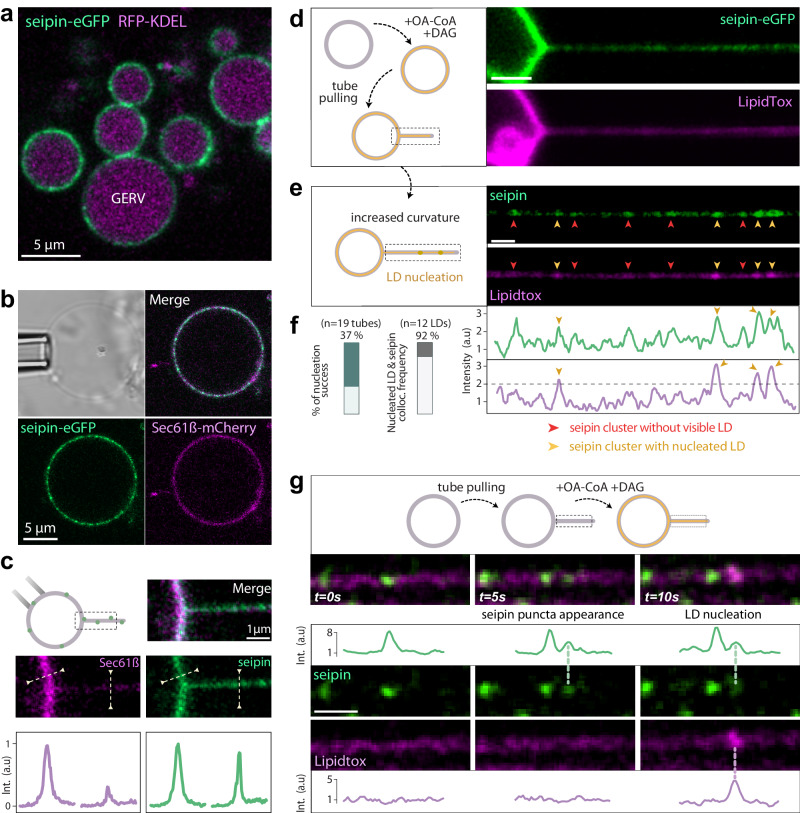
GERVs reproduce lipid droplet assembly. **a** Confocal microscopy image of harvested Giant ER Vesicles (GERVs) containing seipin/KDEL. Scale bar: 5 µm. **b** Confocal microscopy image of an isolated Giant ER Vesicles (GERVs) containing seipin/sec61ß. Scale bar: 5 µm. **c** Top left: Representation of a membrane nanotube extracted from a seipin/sec61ß-based Giant ER Vesicle. The rectangular shape indicates the region of interest. Top right: Confocal microscopy snapshot of the nanotube extracted from the seipin/sec61ß-based Giant ER Vesicle. Middle: Fluorescence profiles drawn perpendicular to the membrane at both the flat region and the nanotube. Intensities are in arbitrary units. **d** Confocal microscopy image of a nanotube extracted from a seipin-based Giant ER Vesicle (GERV), which was fed with OCoA and DAG five minutes before tube pulling. LipidTox fluorescence is used to report for the hydrophobicity of the membrane. **e** The membrane tube shown in (**d**) is pulled to increase its curvature, resulting in the appearance of seipin puncta in the tube. Some seipin puncta are LipidTox-condensed (indicated by yellow arrows), while others lack LipidTox puncta (red arrows). All LipidTox puncta colocalize with seipin spots. Fluorescence profiles indicate the enrichment of LipidTox at specific spots in the tube. **f** Left: fraction of nucleation events observed following tube pulling and curvature increase. A nucleation event is considered when at least one LipidTox-positive puncta forms in the tubule. Right: Frequency of nascent LipidTox-positive nascent LD colocalization with a seipin cluster. **g** Confocal microscopy snapshots of a nanotube extracted from a Giant ER Vesicle (GERV) with seipin overexpressed, and supplied with the substrates for triacylglycerol synthesis. LipidTox fluorescence is monitored to visualize the formation of a nascent LD in the tube. Arrows indicate the appearance of an oil lens (*t* = 10 s) after the formation of a seipin spot (*t* = 5 s).

Seipin-eGFP perfectly localized to GERVs, labeled by RFP-KDEL or sec61ß-mCherry, where it was mobile (Fig. 4a, Supplementary Fig. 6a, b). When we pulled a tubule from the GERV, seipin was much more enriched in the tubule than Sec61ß^17^ (Fig. 4c), indicating that seipin preferred tubules, at least more than Sec61 or LipidTox, serving as a proxy of membrane hydrophobicity (Supplementary fig. 6c).

To demonstrate the ability of GERVs to assemble LDs, we supplemented them with DAG and OA-CoA for TAG synthesis. We used LipidTox to stain TAG in the membrane of the GERVs (Fig. 4d). We then pulled first a tubule and next increased its curvature by rapidly pulling on it further (Fig. 4e, Supplementary Fig. 6d, e). This manipulation resulted in the appearance of LipidTox puncta, at least twice as bright as the background, which perfectly co-localized with seipin (Fig. 4e). We considered these as LD nucleation events, here induced by curvature and controlled by seipin. The induction of curvature seemed also to promote the appearance of more seipin puncta, some of which were negative for nascent LDs (Fig. 4e). Out of 4 independent experiments, 11 out of 12 of the LipidTox-positive nascent LDs were associated with a seipin cluster (Fig. 4f), indicating that seipin controlled the nucleation events. To reinforce our findings, we extracted first a tubule from a GERV and then introduced the substrates for TAG synthesis (Fig. 4g). Throughout our experiment, we observed the progressive development of a seipin puncta, which eventually exhibited a positive response to LipidTox staining within a few seconds (Fig. 4g). Altogether, these data support that seipin controls the condensation of triglycerides into nascent LDs on tubules^41,46–48^.

In addition to observing LD nucleation on tubules, we also encountered situations where LDs appeared on the flat regions of GERVs (e.g. supplementary Fig. 5d). This could be explained by the fact that our substrate-supplying protocol may not fully replicate the cell situation. Indeed, we provided GERVs with both OA-CoA and a significant amount of DAG, which can form droplets by itself and also facilitate the nucleation of TAG LDs^39,49–51^. The presence of such excess DAG during the synthesis of TAG may have contributed to LD nucleation on the flat areas of the GERVs. In support of this interpretation, when GERVs were within cells, feeding only oleic acid resulted in minimal LD formation in these regions of lower curvature^17^. Thus, our data support the notion that TAG LDs may form on flat membrane regions when abnormal amounts of DAG are present alongside TAG.

Together, our above data show that GERVs capture the essential lipid management capacity of the ER. The versatility of GERVs can hence enable further highlighting of biophysical properties and mechanisms of LD biogenesis. Other GOVs may likewise serve to reproduce and study the functionalities of their corresponding organelles^52^.

## Methods

### Cell culture

COS-7, HeLa, and Huh7 cells were maintained in High Glucose (4.5 g/l) with stabilized Glutamine and with Sodium Pyruvate Dulbecco’s modified Eagle’s Medium (DMEM) (Dutscher) supplemented with 10% fetal bovine serum and 1% penicillin/streptomycin (GibcoBRL). Fibroblast cells were maintained in the same conditions but with 1 g/L of glucose. Cells were cultivated 48 h at 37 °C with 5% CO_2_.

To induce feeding conditions and lipid droplet formation (Fig. 3), COS-7 cells were incubated for 24 h with DMEM supplemented with fatty acids conjugated to bovine serum albumin (BSA) (1% vol/vol). Finally, feeding conditions (Fig. 3) were 200 µM of oleic acid; 200 µM of palmitic acid (mixed with ethanol that finally was at 0.2%v/v in final culture media) and 200 µM of oleic acid; 200 µM of linoleic acid and 200 µM of oleic acid; 200 µM of linolenic acid and 200 µM of oleic acid; 200 µM of arachidonic acid and 200 µM of oleic acid. To induce cholesterol-enriched lipid droplet formation, cholesterol was delivered with cyclodextrin. A cholesterol/methyl-ß-cyclodextrin solution was prepared at a final concentration of 1 mM of cholesterol as in the following. A suitable amount of methyl-ß-cyclodextrin was dissolved in cell culture media and then incubated with crystal cholesterol at 1/20 molar ratio (cholesterol/ methyl-ß-cyclodextrin) for 24 h with agitation at 37 °C. The resulting solution was filtered using 0.2 μm syringe filter and conserved at 4 °C until use. To induce starvation conditions (supplementary Fig. 4k), COS-7 cells were incubated in EBSS, calcium, magnesium, and rouge de phenol (ThermoFisher # 24010043).

### Cell transfections and plasmids

Cells were seeded in MatTek 3.5 mm coverslip bottom dishes (MatTek Corp. Ashland, MA) for 16 h before transfections. Cells were transfected with indicated plasmid using jetPEI transfection reagent (PolyPlus #101- 10 N). Cells were transfected with different plasmids fused with fluorescent protein constructs 24 h before giant organelles collection. Here is the list of the used plasmids.

- mCh-Climp63 was a gift from Gia Voeltz (Addgene plasmid # 136293; http://n2t.net/addgene:136293; RRID:Addgene_136293).
- Both RFP-KDEL and P58GFP are a gift from Catherine L. Jackson from Jacques Monod Institute - UMR 7592 CNRS – Paris university.
- Both dGAT1-EGFP and dGAT2-EGFP are gifts from professor Robert YANG School of Biotechnology and Biomolecular Sciences, the University of New South Wales, Sydney, NSW 2052, Australia.
- ERoxBFP was a gift from Erik Snapp (Addgene plasmid # 68126; http://n2t.net/addgene:68126; RRID:Addgene_68126).
- mCh-Sec61ß was a gift from Gia Voeltz (Addgene plasmid # 49155; http://n2t.net/addgene:49155; RRID:Addgene_49155) (Zurek et al., 2011).
- pEGFP Sec22b was a gift from Thierry Galli (Addgene plasmid # 101918; http://n2t.net/addgene:101918; RRID:Addgene_101918) (Petkovic et al., 2014).
- Seipin human EGFP, pSH-EFIRES-B-Seipin-miniIAA7-mEGFP was a gift from Elina Ikonen (Addgene plasmid # 129719; http://n2t.net/addgene:129719; RRID:Addgene_129719) (Li et al., 2019)
- mCherry-TOMM20-N-10 was a gift from Michael Davidson (Addgene plasmid # 55146; http://n2t.net/addgene:55146; RRID: Addgene_55146)
- Mfn2-YFP was a gift from Richard Youle (Addgene plasmid # 28010; http://n2t.net/addgene:28010; RRID:Addgene_28010) (Karbowski et al., 2002).
- Mito-BFP was a gift from Francesca Giordano Institute for Integrative Biology of the Cell(I2BC), CEA, CNRS, Paris-Sud University, Paris-Saclay University, Gif-Sur- Yvette Cedex 91198, France.
- KDE-GFP (Dipeptidyl peptidase IV in which the extracellular domain had been replaced by the GFP sequence to restrict protein localization to the Golgi apparatus) was a gift from Professor Christian Poüs (Paris-Sud University, France)
- mCh-Golgi-7 was a gift from Michael Davidson (Addgene plasmid # 55052; http://n2t.net/addgene:55052; RRID:Addgene_55052)
- pmCherry-2xFYVE was a gift from Harald Stenmark (Addgene plasmid # 140050; http://n2t.net/addgene:140050; RRID:Addgene_140050)
- pMRXIP Lamp1-Venus was a gift from Noboru Mizushima (Addgene plasmid # 89937; http://n2t.net/addgene:89937; RRID: Addgene_89937) (Tsuboyama et al., 2016)
- GPI_2xmCherry was a gift from Salvatore Chiantia (Addgene plasmid # 127812; http://n2t.net/addgene:127812; RRID:Addgene_127812) (Dunsing et al., 2018)
- eGFP-LC3B adenovirus was kindly provided by Sharon Tooze (London Research Institute, UK) and was amplified in QBI-HEK 293 A cells and purified on a cesium chloride gradient.
- pmTurquoise2-Peroxi was a gift from Dorus Gadella (Addgene plasmid # 36203; http://n2t.net/addgene:36203; RRID:Addgene_36203)

### Fluorescent probes

Cell nucleus was detected by Hoechst 33342 Solution (0.1% v/v; Cat# 62249 *ThermoFisher*). Both organelle membranes and lipid droplets were tagged with HCS LipidTox™ Deep Red Neutral Lipid Stain,, (0.1% v/v; Cat# H34477 Thermo fisher); or AUTOdot, (0.1% v/v; Cat# SM1000a abcepta).

### ER fractionation

To measure lipid incorporation in ER membranes in mass spectrometer analysis, we made 3 different conditions. A control condition without lipid incubation; Oleic / Palmitic acid molar ratio 50/50 at 200 µM each; linolenic / linoleic molar ratio 50/50 at 200 µM each. Palmitic acid was dissolved in ethanol that finally was at 0.2%v/v in the final culture media). The mixture then was added to 37 °C heated 10 % BSA in DPBS solution, it was vortexed, sonicated, and incubated at 37 °C with agitation for 1 h then sufficient quantity to get 200 µM final lipid concentration. Finally, it was added to cell culture media used to treat the cells. linolenic / linoleic mixture was added directly to 10 % BSA solution and treated as palmitic / oleic.

COS7 cells were seeded in P150 mm cell culture plate and incubated for 24 h. Cells were then incubated with cell culture media containing the indicated exogenous lipid at 200 µM. After incubation, culture media were removed and the cells were washed twice with DPBS 1X. 1 ml of homogenizing buffer (10 mM Tris/HCl pH 7.4, 1 mM EGTA, 0.5 mM EDTA and 0.25 M sucrose, Complete^TM^ protease inhibitors) was added to cells then they were scraped. Cells were incubated on ice for 10 minutes then they were mechanically lysed with 20 passages within 26 G needle. The cell lysate was then spun at 1000 g for 10 min at 4 °C to pellet cellular debris and nuclei (pellet (Nuclei fraction) and (post nuclei supernatant PNS fraction). PNS fraction was spun at 9000 g to pellet the mitochondria (pellet (mitochondria fraction) and post mitochondria supernatant PMS). PMS was spun at 320000xg for 1 h, three fractions were collected:

PMS fraction was mixed with Iodixanol to yield 1 ml of 35% idoxanol. 900 µl were transferred to 3 ml open thin wall ultracentrifugation tub 900 µl of 20% iodixanol in homogenizing buffer and 900 µl of 10 % iodixanol were layered on top of the lysate/iodixanol solution. Top layer of 100 µl of 0% iodixanol was added and the gradient was spun at 320,000 g for 1 h at 4 °C in a TST60 rotor. Ten fractions of 280 µl were collected from the top (fraction 1). Fractions were analyzed by WB as following: 1 volume of NuPAGE LDS Sample Buffer (Thermo Fisher Cat: NP0007) was added to 3 volumes from each fraction and then heated at 95 °C for 7 minutes. The proteins were separated on SDS–PAGE and electro-transferred onto a nitrocellulose membrane. After transfer, the membrane was saturated in DPBS containing 0.1% Tween 20 and 5% milk. Primary antibodies were added overnight at 4 °C or for 2 h at room temperature depending on the antibody. The membranes were washed with DPBS containing 0.1% Tween and incubated for 1 h at room temperature with appropriate HRP-conjugated secondary antibody. ECL plus kit (Thermo Scientific Cat: 32132) was used for protein detection. The quantity of proteins in ER fractions was analyzed by the Coomassie (Bradford) Protein Assay Kit (thermoFicher # 23200).

### GOVs production and extraction

After transfection, the cultured cells were incubated for 24 h to 70-80% confluence. Then, they were transferred into a hypotonic culture media DMEM: H2O (5:95% v/v) at pH 7.4, at 37 °C, 5% CO2, to induce GOVs^17^. Confocal microscopy Z-stacks were acquired on entire swollen cells to be able to measure GOVs’ diameter distribution (Fig. 1, supplementary Fig. 1). We only considered organelles vesicles over 0,75 µm in diameter in the size distribution measurements and representation. Size distributions are shown as frequency distributions by using GraphPad Prism software and the violin plot settings (Fig. 1b) or scatter plots (supplementary Fig. 1h).

A swollen cell was caught with a micropipette and aspired until the breakage of its plasma membrane, due to the reach of its lysis tension. GOVs in the cell were subsequently released and captured by another pipette (Fig. 1, supplementary Fig. 1a). For more GOV recovery, the swollen cells were subjected to gentle pipetting to shear the cells (pipette with an inner diameter of 1.2 mm), with at least four suction and refill cycles. This process lysed the cell plasma membrane and led to the release of GOVs.

### Nocodazole treatment

To induce larger GOVs (supplementary Fig. 1i, j), COS-7 cells were incubated with a culture medium containing nocodazole (Cat#487928 from Calbiochem) (2.5 μg/ml) for 1 hour at 37 °C. Cells were then imaged after swelling in the hypotonic media.

### Micro-manipulation

Micropipettes were used to manipulate cells and GOVs. Micropipettes were made from capillaries (1.0 OD, 0.58 ID, 150 L (mm)), 30-0017 GC100-15b; Harvard Apparatus, Holliston, MA) with a micropipette puller (model P-2000; Sutter Instruments). The pipettes were subjected to plasma cleaner and treated with a PEG-based solution (Cat #JKA3037 from Merck) at 3 mg/mL in ethanol: H_2_O (95:5 v/v) solution. Then, micro-pipettes were cleaned into DMEM: H_2_O (5:95 v/v). Micromanipulation robot (TransferMan 4r) was provided by Eppendorf (Hamburg, Germany). Before micromanipulation, biological samples were injected on a cover-slip glass plate, pre-treated with BSA, and cleaned with DMEM: H_2_O (5:95 v/v).

### GOVs membrane tension measurements

Using Laplace’s law, and the measurement of the pipette inner radius (R_p_), GOV radius (R_GOV_), and suction pressure $\Delta P$, the surface tension $\gamma$ of the interface was calculated as follows:

1 $$\gamma=\frac{\Delta P.{R}_{p}}{2(1-{R}_{p}/{R}_{{GOV}})}$$

See supplementary Fig. 4g, h.

The suction was carried out using a syringe. The resulting pressure was measured with a pressure transducer (DP103; Validyne Engineering, Northridge, CA). The output voltage was monitored with a digital voltmeter (FLUKE 16 multimeter) which gave us a pressure measurement sensibility of 1 Pa. The pressure transducer was calibrated before the experiments.

### GOVs lysis tension measurements

We used the micropipette aspiration technique and a manometer (PCE P15) to measure the suction pressure $\Delta P$ (1) during GOV’s lysis tension measurements. Thanks to a slight aspiration, a bilayer tongue was first sucked into the micropipette. The aspiration was then increased at a constant rate of approximately 10mbar/min, causing a proportional increase in the bilayer surface tension (see the section below). At a certain tension, the GOV ruptured because of a pore opening in its membrane. The lysis tension was taken as the higher tension reached just before bilayer rupture.

### GOVs apparent area expansion modulus measurements

GOVs were gently captured with a micropipette. Then, aspiration in the micropipette was slowly increased to obtain multiple values of membrane tension (only for membrane tension above 0,75mN/m, ~ 20 seconds were let between each increment of tensions to reach equilibrium). For each value of applied bilayer tensions, variation of both membrane tongue length (in the micropipette) and radius of the GOV were measured (supplementary Fig. 2g, h). This allowed determining both the exact surface area ${A}_{\gamma }$ (2) of a GOV for a given membrane tension $\gamma$ and its variation of area $\Delta {A}_{\gamma }$ (3) between the initial membrane tension ${\gamma }_{0}$ and the current one $\gamma$^53^:

2 $${A}_{\gamma }=2{{{{{\rm{\pi }}}}}}.\left({R}_{{GOV},\gamma }^{2}\left[1+\sqrt{1-{({R}_{p}/{R}_{{GOV}},\gamma )}^{2}}\,\right]+{R}_{p}.{L}_{p,\gamma }\right)$$

And,

3 $$\Delta {A}_{\gamma }=	 2\pi \left({R}_{{GOV},\gamma }^{2}\left[1+\sqrt{1-{({R}_{p}/{R}_{{GOV}},\gamma )}^{2}}\,\right]\right. \\ 	 \left. {-R}_{{GOV},{\gamma }_{0}\,}^{2}\left[1+\sqrt{1-{({R}_{p}/{R}_{{GOV}},{\gamma }_{0})}^{2}}\,\right]+{R}_{p}\left({L}_{p,\gamma }-{L}_{p,{\gamma }_{0}}\right)\right)$$

Where ${L}_{p,\gamma }$, ${L}_{p,{\gamma }_{0}}$, R_p_, ${R}_{{GOV},\gamma }$ and ${R}_{{GOV},{\gamma }_{0}}$ are respectively the bilayer tongue length in the micropipette for the initial tension ${\gamma }_{0}$ and a given tension $\gamma$, the inner pipette radius, and the GOV radius both at tension $\gamma$ and initial tension ${\gamma }_{0}$. The applied bilayer tension $\gamma$ was then plotted against the relative surface area variations of the GOV’s bilayer $\Delta {A}_{\gamma }/{A}_{\gamma,0}$ (supplementary Fig. 2i–k). For tensed membrane regimes (tension above 0.75mN/m), membrane fluctuations were negligible and the bilayer tension $\gamma$ can be linked to the surface area variation $\Delta {A}_{\gamma }/{A}_{\gamma,0}$ by the apparent area expansion modulus (${\alpha }_{{expansion}}$):

4 $${\alpha }_{{expansion}}=\frac{\gamma }{\Delta {A}_{\gamma }/{A}_{\gamma,0}}$$

Therefore, the slope of the bilayer tension against the area relative variation, extracted with linear regression, gave us the apparent area expansion modulus of the membrane (${\alpha }_{{expansion}}$).

### GOVs bending modulus measurements

To determine the bending moduli, we used the same method as in Rawicz & al., 2000. Like in the previous section, we determined area variations of GOVs for different membrane tensions, but, in the range of minuscule bilayer tensions $\gamma$ < 0.5mN/m. To do so, we especially had to decrease the initial membrane tensions of GERVs and GMVs, by putting them in contact with a sucrose solution to reach a final osmolarity of 40 mOsm/L. Then, the GOVs were caught and we spanned three orders of magnitudes of membrane tensions (0.001 mN/m to 0.1mN/m). In these regimes, vesicle area relative variations increase linearly with ln($\gamma$) (supplementary Fig. 2s). The slope $\lambda$ of this linear curve was obtained thanks to linear regression for ln($\gamma$) with values which were between −6 and −2. Then, $\lambda$ was multiplied by ${K}_{B}T/8\pi$ (where ${K}_{B}$ and T are respectively the Boltzmann constant and the temperature) to obtain the bending moduli $\kappa$ (supplementary Fig. 2t).

### Sampling of GOVs for biophysical properties

Our GOV samples for membrane properties measurements were chosen with initial radius between (2 µm and 12 µm). To reduce the uncertainty of our measurements, we measured membrane properties only for GOVs which were following this ratio: ${R}_{p}/{R}_{{GOV}} < 0.5$ (where R_p_ is the pipette inner radius and R_GOV_ the GOV radius). Note that for Fig. 1d–g, mCherry-TOMM20-N-10 based GMVs, RFP-KDEL based GERVs, P58GFP based GGVs, pMRXIP Lamp1-Venus based GLVs, pmCherry-2xFYVE based GEEVs, and GPI_2xmCherry based GPMVs were used to measure biophysical parameters. Note that for GERV-contacts (Fig. 2a–c), only KDEL or ERox-based GERVs (bulk marker) exhibiting contact with at least two other organelles of the same type were chosen. For Fig. 2 and supplementary Fig. 2k, only KDEL-based GERVs were used to measure biophysical properties.

### Lipid supply to GERVs

A first supply mixture of diolein (Cat# D0138 from Merck) at 8 mM were mixed with BSA (0,5%) in hypotonic culture media (DMEM: H_2_O 5:95) by vortexing three times during 10 seconds. A second supply mixture of 18:1 (n9) CoenzymeA (Cat# 870719 P from Avanti) at 1 mM and 18-NBD 18:1 Coenzyme A (Cat# 810229 from Avanti) at 20 µM was prepared in hypotonic culture media (DMEM: H_2_O 5:95). GOVs were incubated for 1 hr at 37 °C and 5% CO_2_ and supplied with these two mixtures at 2.5% v/v each. Finally, final concentrations are 200 µM, 25 µM, and 0,5 µM respectively for the diolein, OCoA, and NBD-OCoA. The incorporation of OCoA into triglycerides in GERV has been monitored thanks to the fluorescence signal of NBD-OCoA (Fig. 3a, supplementary Fig. 5a–d) and mass spectrometry analysis. Snaps were taken every 2 minutes and the fluorescence was analyzed by drawing an orthogonal line profile (10 pixels thick) to the GERV membrane, always at the same position. Then, each peak of fluorescence was registered (Fig. 3a). Same quantifications were made (Fig. 3b). After 1 hr of OCoA supply, the hydrophobicity of GERVs’ membrane was quantified thanks to LipidTox addition (0.05% v/v).

### TAG-NBD synthesis in GERV, lipid extraction, TLC analysis and MS analysis

To quantify the presence of TAG-NBD synthesis in GERV membrane, we performed the lipid supply to GERVs as above section by using these following mixtures: A first supply mixture of diolein (Cat# D0138 from Merck) at 8 mM were mixed with BSA (0,5%) in hypotonic culture media (DMEM: H_2_O 5:95) by using a vortex. A second supply mixture of 18-NBD 18:1 Coenzyme A (Cat# 810229 from Avanti) at 1 mM was prepared in hypotonic culture media (DMEM: H_2_O 5:95). After extraction, GOVs were incubated for 1 hr at 37 °C and 5% CO_2_ and supplied with these two mixtures at 2.5% v/v each. Finally, final concentrations are 200 µM and 25 µM respectively for the diolein and NBD-OCoA. After one hour of incubation, all vesicles were placed at 4 °C to stop the reaction of TAG-NBD synthesis. Then, the media were centrifuged at 300000 g for 30 minutes to isolate all the membranes at the pellet.

Organelle pellets and supernatants were extracted by a modified Folch extraction (J Biol Chem. 1957;1:497–509). Briefly, GOVs (extracted from 10^6^ cells) and 1 ml of supernatant were homogenized with chloroform/methanol (2/1) to a final 5 ml of solvent mixture. After dispersion, the whole mixture was agitated during 15-20 min in an orbital shaker at room temperature. The homogenate was centrifuged to recover the liquid phase. The solvent was washed with 1 ml of water. After vortexing some seconds, the mixture is centrifuged at low speed (2000 rpm) to separate the two phases and the lower phase was recovered. The lower chloroform phase containing lipids was evaporated under a nitrogen stream. The lipid extract was re-suspended in 1 ml of chloroform/methanol (2/1) and transferred to a 1,5 ml Eppendorf and dry under a nitrogen stream. The lipid extract was then re-suspended in 100 µL of chloroform/methanol (2/1).

20 µl of each lipid extract were loaded on a pre-eluted 10×10 cm HPTLC Silica gel 60 plate (Supelco). TLC was eluted with 60:40:1 Chl:MeOH:water. After elution, the TLC was dried, and the TLC was exposed to 405 nM UV light and a picture was taken with a digital camera.

For MS analysis, we mixed 10 µl of the extracts with 10 µl of propanol:methanol:chloroform (4:2:1) and infused in a QExactive Plus (Thermo Fisher Scientific) with a robotic infusion source Triversa Nanomate (Advion). NBD-TAG was detected by FTMS (Resolution= 280000 at m/z = 200) in negative ion mode as a deprotonated anion relying on the accurate mass measurement. For further validation and to check for the putative incorporation of NBD-oleoyl into other lipid classes, we established the fragmentation of NBD-TAG standard. We then acquired a data independent analysis of the lipid extracts where we fragmented all the masses from 590-1200 Da. Acquisition was performed in negative ion mode with 2 microscans at the resolution of 17500 (m/z = 200), AGC was set to 1e5 and maximum IT was 64 ms. Isolation window was set to 1 Da, first m/z was 150 Da, NCE was set to 35 and data was acquired in centroid mode. We then probed the data for the formation of the specific fragment from oleoyl-NBD m/z = 378.2551 corresponding to the sum composition C24H32N3O.

### Nanotube extraction from GERVs and protein partitioning

Nanotubes were pulled from GERVs thanks to a micropipette. The GOV was gently captured by one of the two micro-pipettes. The over micropipette was slowly moved toward the GOV. Upon contact, some PEG silane coverture defects on the micropipette glass surface allowed the sticking of the GERV membrane. Finally, after adsorption of the GERV membrane on the pipette, a nanotube was extracted from the GERV by removing slowly the micropipette in the opposite direction of the GOV. Fluorescence profiles (10 pixels of thickness) were drawn perpendicular to the GERV membrane and the extracted nanotube (Fig. 4g) to quantify protein partitioning between flat and curved regions.

### Lipid droplet nucleation in GERVs

After GERV extraction, LipidTox was added (0.05% v/v) in the solution to visualize the hydrophobic region in the membrane to report for neutral lipids condensation. Then, on one hand, nanotubes were pulled from seipin-based GERVs thanks to a micropipette and then GERVs were supplied with lipids. Seipin-EGFP and LipidTox signals are monitored in the nanotube thanks to live confocal microscopy. The nucleation of a droplet and its interaction with seipin is finally visualized in the nanotube thanks to the LipidTox signal (Fig. 4d–f, supplementary Fig. 6d, e). Fluorescence profiles (5 pixels of thickness) were drawn perpendicular to the extracted nanotube (Fig. 4e–f) to quantify seipin enrichment where an oil lens has nucleated. On the other hand, following the lipid supply protocol in seipin-based GERVs (section above). Thanks to the nanotube extraction protocol, a curvature is induced on the GERV membrane. Seipin-EGFP and LipidTox signals are monitored in the nanotube thanks to live confocal microscopy. The nucleation of an oil nano-droplet and its interaction with seipin is finally visualized in the nanotube thanks to the LipidTox signal.

### ER-like Giant Unilamellar Vesicles formation

Giant unilamellar vesicles (GUVs) were composed of 40 mol% POPC, 20 mol% DOPC, 10 mol% DOPE, 20 mol% DPPC, 5 mol% DOPI, 5 mol% cholesterol and 0.5% Cy5 DOPE (Avanti polar lipids, Inc). GUVs were prepared by electroformation at 35 °C and imaged at room temperature. PLs and mixtures thereof in chloroform at 2.5 mM were dried on an indium tin oxide (ITO) coated glass plate. The lipid film was dessicated for 1 h. The chamber was sealed with another ITO-coated glass plate. The lipids were then rehydrated with a sucrose solution (60 ± 5 mOsm). Elec- troformation was performed using 100 Hz AC voltage at 1.4 Vrms and maintained for at least 2 h. This low voltage was used to avoid hydro- lysis of water and dissolution of the titanium ions on the glass plate. GUVs were directly collected with a Pasteur pipette.

### Confocal microscopy imaging

All micrographs were made on a Carl ZEISS LSM 800. GFP fluorescence was excited at 488 nm, and emission was detected between 510 and 550 nm, while mCherry tagged protein fluorescence was excited at 540 nm, and emission was detected between 580 and 650 nm. Deep red fluorescence was excited at 640 nm and was detected above 650 nm. BFP fluorescence was excited at 420 nm and detected below 500 nm. All fluorescence signals were analyzed with ImageJ.

### FRAP experiment in GERVs

Fluorescence recovery after photobleaching (FRAP) experiments were performed by bleaching a part of the GOV bilayer (Supplementary Fig. 6a). Fluorescence proteins were bleached, and then, the recovery of both signals was monitored. The FRAP curves were normalized by the fluorescence before bleaching GOVs and just after the bleach in the region of interest. To avoid losses of signals during FRAP, we re-normalized our recovery curve with the fluorescence signals in the non-bleached regions of the GOV. GraphPad Prism was used to fit the FRAP recovery curve with a nonlinear regression and the exponential “one-phase association model.”.

### Statistical analysis (see source data)

#### Data Sets

Sample analysis (sample size, test details, descriptive statistics, data sets) are available in the Source Data.

### Data representation

When box graphs are plotted (Figs. 1d–g, 2c, e-g, 3c, e and supplementary Fig. 2v, 4k), the middle line represents the median value of the sample. The color box extremities represent the first and the third quartile of the whole sample (n). The mean of each independent replicate (N) is shown with larger points on the plot. All the data points (n) are shown with small and transparent points on the graphs, revealing the whole distribution of the sample. When scatter plots were plotted, values shown in the text and Figures are mean and standard deviation (SD). The number of independent measurements are indicated in captions of principal figures and the Source Data. When “N” is used, it refers to N different day of analysis. For all measurements of the physical properties of GOVs, each data point refers to GOVs extracted from independent cells. Size distributions are shown as frequency distributions by using GraphPad Prism software and the violin plot settings (Fig. 1b and supplementary Fig. 1m, p) or scatter plots (supplementary Fig. 1h, k, l, n, o).

### Statistical tests

The statistical analyses were made with GraphPad Prism 7.0a. For the statistical analysis of the data plotted in supplementary Fig. 1h, k, n, each data set distribution was first submitted to a Shapiro-Wilk Normality test to control sample Gaussian distributions. Distributions were non-Gaussians (*P* > 0.05), and samples were compared with a parametric unpaired t-test with Welch’s correction. For statistical analysis of data plotted in (Figs. 1d–g, 2c, e–g, and supplementary Fig. 2v, 4c, k), we performed a nested One-Way ANOVA - Multiple comparisons: Fisher LSD test. Individual P values are shown on the plots. For statistical analysis of data plotted in Fig. 3c, e, we performed a nested t-test with two-tailed P-values, which are shown in the figures. For supplementary Fig. 4e, g, we performed an unpaired t-test with a two-tailed *P* value (each single point is an independent data).

### Reporting summary

Further information on research design is available in the Nature Portfolio Reporting Summary linked to this article.

### Supplementary information

Supplementary Information

Reporting Summary

Supplementry data
