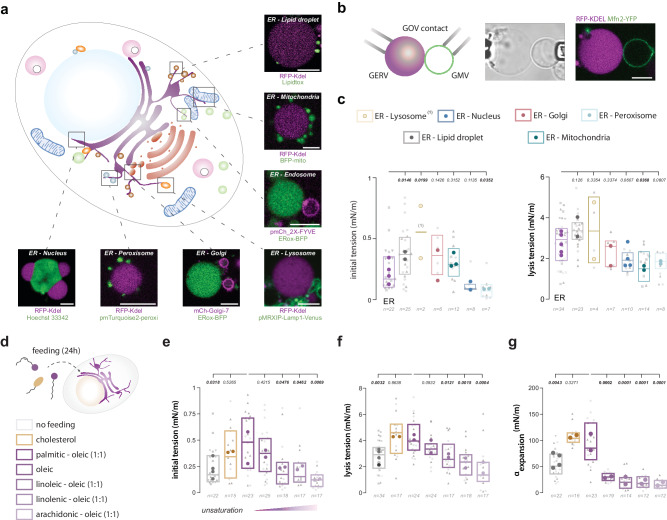# Author Correction: Giant organelle vesicles to uncover intracellular membrane mechanics and plasticity

**DOI:** 10.1038/s41467-024-49026-1

**Published:** 2024-06-07

**Authors:** Alexandre Santinho, Maxime Carpentier, Julio Lopes Sampaio, Mohyeddine Omrane, Abdou Rachid Thiam

**Affiliations:** 1 Laboratoire de Physique de l’École normale supérieure, ENS, Université PSL, CNRS, Sorbonne Université, Université Paris Cité, F-75005 Paris, France; 2 Institut Curie, PSL Research University, Plateforme de Métabolomique et Lipidomique, 26 rue d’Ulm, Paris, France

**Keywords:** Membrane biophysics, Biophysical methods, Organelles

Correction to: *Nature Communications* 10.1038/s41467-024-48086-7, published online 04 May 2024

The original version of this article contained an error in Figure 2, in which the y-axis label of the graph in panel f was inadvertently omitted. This has been corrected in both the PDF and HTML versions of the article.