# Supplementary material for: Giant organelle vesicles to uncover intracellular membrane mechanics and plasticity
Source: Nat Commun. 2024 May 4;15:3767. doi: 10.1038/s41467-024-48086-7 (PMC11069511; doi:10.1038/s41467-024-48086-7)
Supplement: Supplementary file 1 — Supplementary Information [file 41467_2024_48086_MOESM1_ESM.pdf]

Giant Organelle Vesicles to Uncover Intracellular Membrane Mechanics and Plasticity

Alexandre Santinho[1], Maxime Carpentier[1], Julio Lopes-Sampaio[2], Mohyeddine Omrane[1], Abdou Rachid Thiam[1]*

[1] Laboratoire de Physique de l'École normale supérieure, ENS, Université PSL, CNRS, Sorbonne Université, Université Paris Cité, F-75005 Paris, France
[2] Institut Curie, PSL Research University, Plateforme de Métabolomique et Lipidomique, 26 rue d'Ulm, Paris, France

*Correspondance to :
Abdou Rachid Thiam
Laboratoire de Physique Statistique,
Ecole Normale Supérieure,
PSL Research University,
75005 Paris Cedex 05, France
thiam@ens.fr

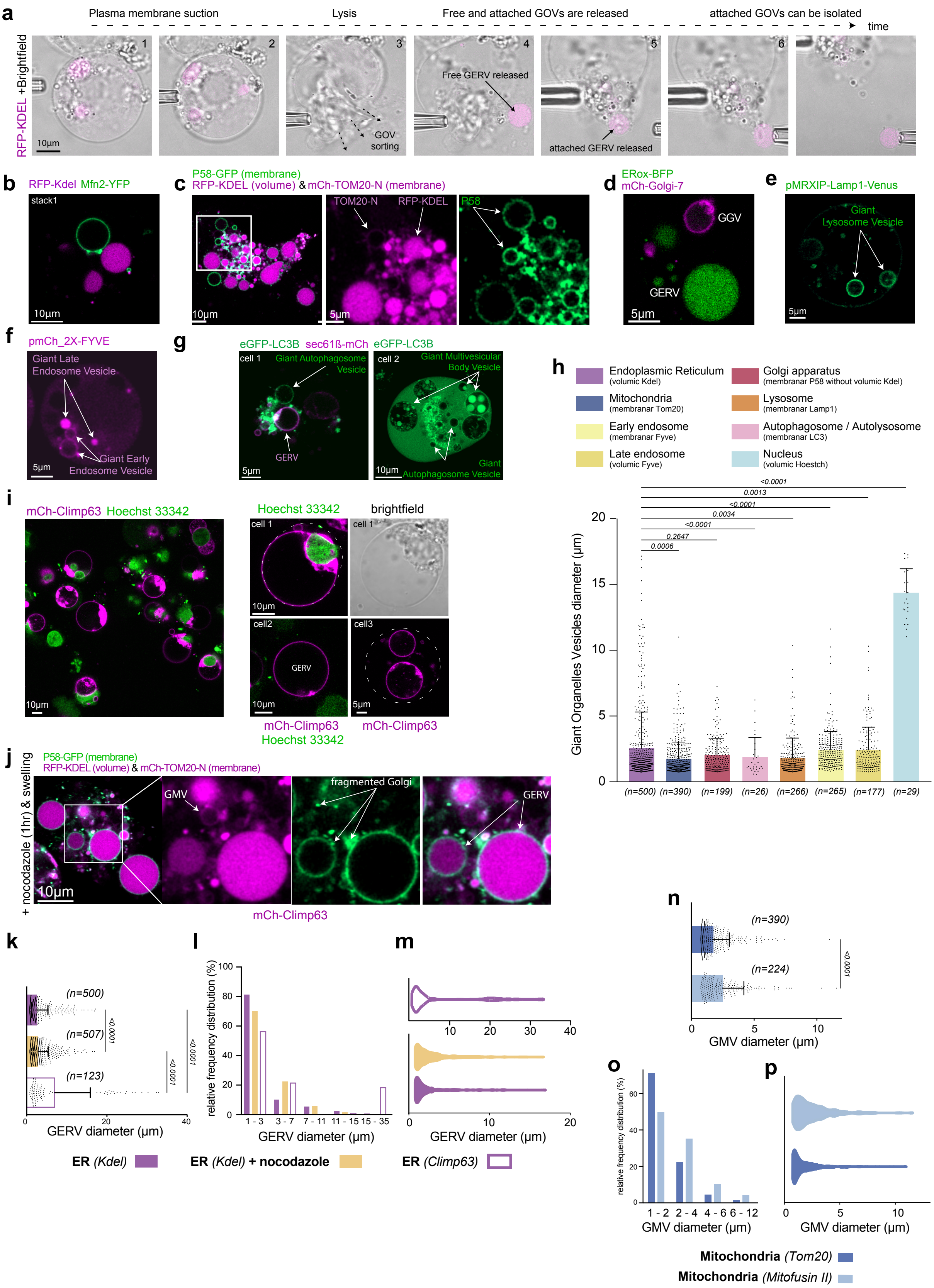

Supplementary Figure 1, related to Figure 1

a. Confocal microscopy image of harvested GERVs containing RFP-KDEL. Scale bar: 5µm.

b. Confocal microscopy image of a swollen COS-7 cell overexpressing RFP-KDEL (luminal marker of ER) and Mfn2-YFP (membrane marker of mitochondria), demonstrating the formation of Giant ER Vesicles (GERVs) and Giant Mitochondria Vesicles (GMVs).
c. Confocal microscopy image of a swollen COS-7 cell overexpressing RFP-KDEL (volume marker of GERVs), mCh-TOMM20-N (membrane marker of GMVs), and the cargo P58-GFP (labeling Giant Golgi Vesicles, GGVs, and GERVs). The image illustrates the presence of GERVs and GGVs.
d. Confocal microscopy image of swollen cells overexpressing ERox-BFP (reporting GERVs) and mCherry-Golgi-7 (reporting GGVs).
e. Confocal microscopy image of a swollen COS-7 cell overexpressing pMRXIP-Lamp1-Venus, visualizing Giant Lysosome Vesicles (GLVs).
f. Confocal microscopy image of a swollen COS-7 cell overexpressing pmCh_2X-FYVE, demonstrating the formation of Giant Early Endosome Vesicles (GEEVs) with a membrane fluorescence signal and Giant Late Endosome Vesicles (GLEVs) with luminal fluorescence signal.
g. Confocal microscopy image of swollen COS-7 cells overexpressing eGFP-LC3B and/or sec61ß-mCh under starvation conditions. The images show puncta of eGFP-LC3B in one cell, Giant Autophagosome Vesicles (GAVs) positive for eGFP-LC3B on the membrane in another cell, and GAVs and Giant Multivesicular Body Vesicles (GMBVs) in a third cell.
h. Top: Different types of GOVs with their size distribution analyzed in the bottom panel (related to Figure 1B). Each point represents a single vesicle, and the number of analyzed vesicles (n) per sample is indicated at the bottom of the plot. Each condition was repeated N=2 times, independently. Each sample was first submitted to a Shapiro-Wilk Normality test to control sample Gaussian distributions. Distributions were non-Gaussians ($P > 0.05$), and samples were compared with a parametric unpaired t-test with Welch's correction. P-values are indicated.
i. Confocal microscopy image of Climp63-based GERVs in swollen cells overexpressing mCh-Climp63. Left: Overview of the cells with their nucleus (labeled with Hoechst 33342) and displaying large GERVs, exceeding 20µm in diameter. Right: other zoomed examples.
j. Confocal microscopy image of swollen COS-7 cells pre-treated with nocodazole for 1 hour. Left: Cells overexpressing RFP-KDEL, mCh-TOMM20-N, and P58-GFP, showing fragmented Golgi and large GERVs.
k. Histogram of the average GERV diameter in swollen COS-7 cells for the conditions studied in panels i, j, as well as the control condition. Mean +/- standard deviation; n represents the number of analyzed vesicles for each condition. Each condition was repeated N=2 times independently. Each sample was first submitted to a Shapiro-Wilk Normality test to control sample Gaussian distributions. Distributions were non-Gaussians ($P > 0.05$), and samples were compared with a parametric unpaired t-test with Welch's correction. P-values are indicated.
l. Histogram of the relative frequency distribution of GERVs in swollen COS-7 cells for the conditions studied in panels i, j, as well as the control condition. The nocodazole-treated condition shows larger GERVs (in the 3-7µm range) compared to the control.
m. Violin plot of the frequency distribution of GERV diameter in swollen COS-7 cells for the conditions studied in panels i, j, as well as the control condition.
n. Histogram of the diameter of GMVs based on mCh-TOMM20-N and Mfn2-YFP fluorescence signals. Mean +/- standard deviation; n represents the number of analyzed vesicles for each condition. Each condition was repeated N=2 times independently. Each sample was first submitted to a Shapiro-Wilk Normality test to control sample Gaussian distributions. Distributions were non-Gaussians ($P > 0.05$), and samples were compared with a parametric unpaired t-test with Welch's correction. P-values are indicated.
o. Histogram of the relative frequency distribution of GMV diameter based on mCh-TOMM20-N and Mfn2-YFP fluorescence signals.
p. Violin plot of the diameter distribution of GMVs based on mCh-TOMM20-N and Mfn2-YFP fluorescence signals. (Fig. k, n: Mean +/- standard deviation; See Supplementary Data Table 4 for statistical analysis).

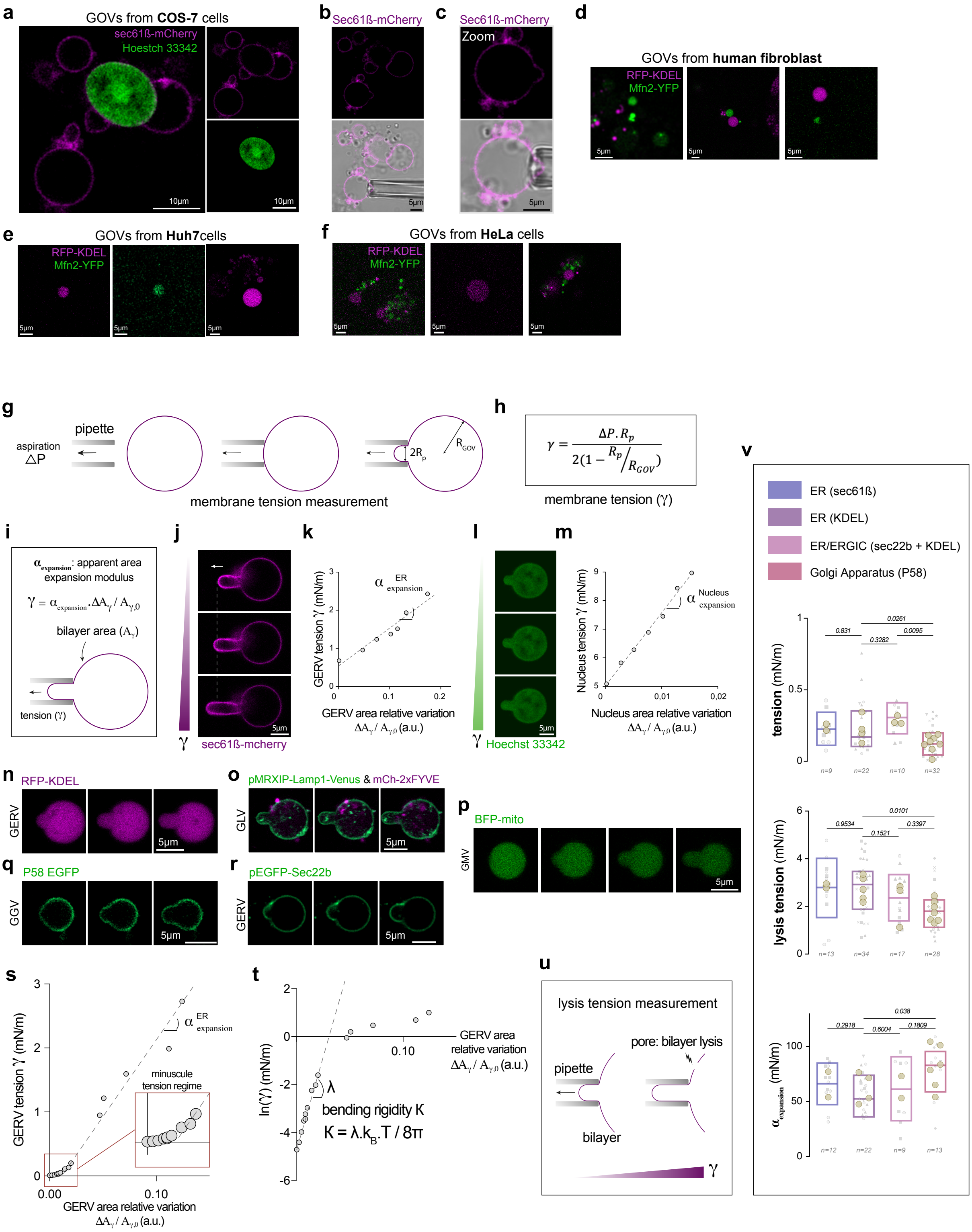

Supplementary Figure 2, related to Figure 1 and Figure 2

a. Confocal microscopy image of sec61ß-GERVs (nucleus labeled with Hoechst 33342) extracted from COS-7 cells.

b. Sec61ß-positive GERVs are isolated using a micropipette and zoom-in in c.

d. Confocal microscopy image of KDEL-based GERVs and Mfn2-based GMVs produced from fibroblast cells.
e. Confocal microscopy image of KDEL-based GERVs and Mfn2-based GMVs produced from Huh7 cells.
f. Confocal microscopy image of KDEL-based GERVs and Mfn2-based GMVs produced from HeLa cells.
g. Schematic representation of the protocol for measuring membrane tension. The micropipette is gradually brought close to the GOV until a gentle aspiration of a membrane tongue occurs. The initial tension of the membrane can be measured when the length of the tongue in the pipette is equal to the radius of the pipette. The membrane tension (γ) of a GOV is determined using two Laplace's laws and the equation shown (h), taking into account the inner radius of the pipette (Rp), the radius of the GOV (RGOV), and the suction pressure (ΔP).
i. Schematic representation of the protocol for measuring the apparent area expansion modulus ($\alpha_{expansion}$). The membrane tension is gradually increased, and for each increment of tension, the variations in GOV surface area are recorded.
j. Measurement of $\alpha_{expansion}$ on a sec61ß-based GERV using a micropipette and confocal microscopy.
k. Plot of membrane tension against the relative area variation of the GERV. The slope of the dotted line (linear regression curve) corresponds to the apparent area expansion modulus of the GERV.
l. Measurement of $\alpha_{expansion}$ on a nucleus.
m. Plot of membrane tension against the relative area variation of the nucleus. The slope of the dotted line (linear regression curve) corresponds to the apparent area expansion modulus.
n. Measurement of $\alpha_{expansion}$ on a KDEL-based GERV.
o. Measurement of $\alpha_{expansion}$ on a Lamp1-based GLV.
p. Measurement of $\alpha_{expansion}$ on a Mito-based GMV.
q. Measurement of $\alpha_{expansion}$ on a P58-based GGV.
r. Measurement of $\alpha_{expansion}$ on a Sec22b-based GERV.
s. Bending rigidity measurement. The plot of membrane tension (on a logarithmic scale) against the area relative variation of a GERV. The early tension regime (<0.5mN/m) allows the determination of the bending rigidity.
t. Plot of ln(γ) against the area relative variations of a GERV (same data points as in m). For ln(γ) values between -6 and -1, the slope of the linear curve (linear regression curve on the minuscule tension regime) is extracted from the graph. The equation used to compute the bending rigidity of GOVs is displayed in the plot.
u. Illustration of lysis tension measurements. The membrane tension is gradually increased at a constant rate until the membrane breaks. The tension recorded just before the membrane breaks corresponds to the lysis tension.
v. Top. Plot of the initial membrane tensions of sec61ß-based GERVs, KDEL-based GERVs, (KDEL/Sec22b)-based GERVs, and P58-based GGVs. For organelles from left to right, the number of independent experiments is N=2; N=4; N=3; N=8; Each experimental data point corresponds to a GOV from a different cell, and the mean of each replicate is shown. Nested One-Way ANOVA - Multiple comparisons: Fisher LSD test. Individual P values are shown. Middle. Plot of the lysis tensions of sec61ß-based GERVs, KDEL-based GERVs, (KDEL/Sec22b)-based GERVs, and P58-based GGVs. For organelles from left to right, the number of independent experiments is N=2; N=5; N=3; N=7; Each experimental data point corresponds to a GOV from a different cell and the mean of each replicate is shown. Nested One-Way ANOVA - Multiple comparison: Fisher LSD test. Individual P values are

shown. Bottom: Plot of the elastic modulus of sec61ß-based GERVs, KDEL-based GERVs, (KDEL/Sec22b)-based GERVs, and P58-based GGVs. For organelles from left to right, the number of independent experiments is N=2; N=4; N=2; N=6; Each experimental data point corresponds to a GOV from a different cell and the mean of each replicate is shown. Nested One-Way ANOVA - Multiple comparison: Fisher LSD test. Individual P values are shown.
(Fig v; Median, 1st and 3rd quartiles. See Supplementary Data Table 5 for statistical analysis and data sets).

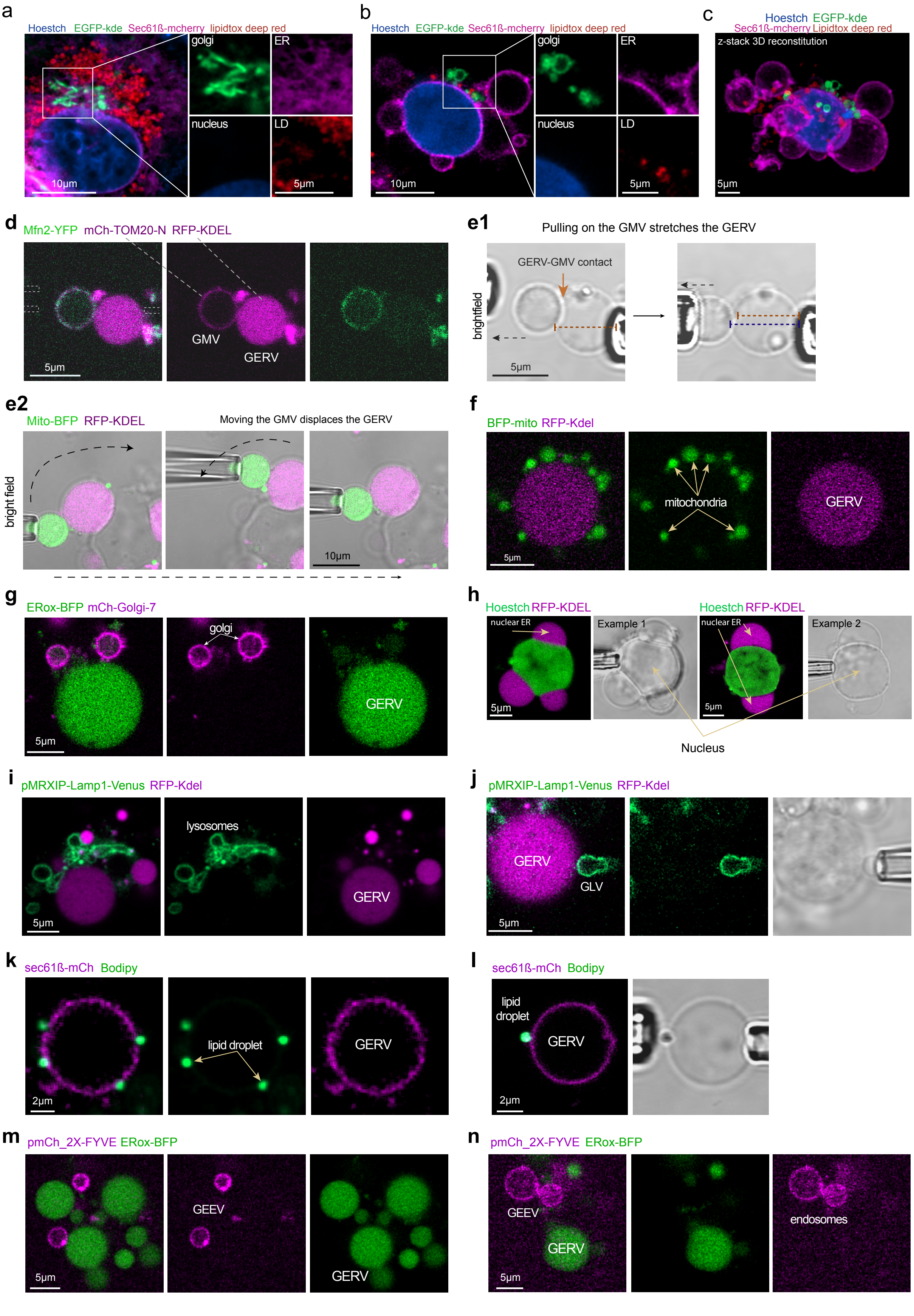

Supplementary Figure 3, related to Figure 2

a. Confocal microscopy image of a COS-7 cell overexpressing sec61ß-mCh and KDE-GFP. Hoechst 33342 and LipidTox Deep Red allowed us to visualize the nucleus and lipid droplets, respectively.
b. Confocal microscopy image of a swollen COS-7 cell overexpressing both sec61ß-mCh and KDE-GFP, in the presence of Hoechst 33342 and LipidTox Deep Red.
c. 3D reconstruction of a Z-stack acquired from a swollen COS-7 cell overexpressing both sec61ß-mCh and KDE-GFP, in the presence of Hoechst 33342 and LipidTox Deep Red. Multiple organelles exhibiting multipartite contacts can be observed. The 3D reconstruction was performed using the 3D viewer plugin in ImageJ.
d. A Mito-based GMV in contact with a KDEL-based GERV. Moving the micropipette holding the GMV moves the GERV and the entire structure, indicating a strong contact between the GMV and the GERV.
e1. A brightfield image of the same contact is shown. The red dashed line indicates the initial diameter of the GERV. Right: Pulling on the GMV with another pipette stretches the GERV due to the strong contact. The blue line represents the diameter of the pulled GERV, larger than the initial diameter.
e2. A GMV is in contact with a KDEL-based GERV, which is also in contact with the nucleus. Moving the micropipette holding the GMV directly moves the GERV and the entire structure, indicating strong contacts.
f. Confocal microscopy image of KDEL-based GERVs in close contact with multiple GMVs.
g. Confocal microscopy image of ERox-based GERVs in close contact with Golgi7-based GGVs.
h. Confocal microscopy image of KDEL-based GERVs in close contact with the nucleus, visualized using Hoechst 33342.
i. Confocal microscopy image of KDEL-based GERVs in close contact with multiple Lamp1-based GLVs.
j. Confocal microscopy image of KDEL-based GERVs in close contact with a GLV held by a micropipette.
k. Confocal microscopy image of sec61ß-based GERVs in close contact with lipid droplets, visualized using LipidTox.
l. Confocal microscopy image of sec61ß-based GERVs in close contact with a lipid droplet, visualized using LipidTox.
m. Confocal microscopy image of an ERox-based GERVs in contact with a FYVE-based GEEVs.
n. Confocal microscopy snapshot of ERox-based GERVs not in contact with FYVE-based GEEVs. These contacts were rarer, difficult to find.

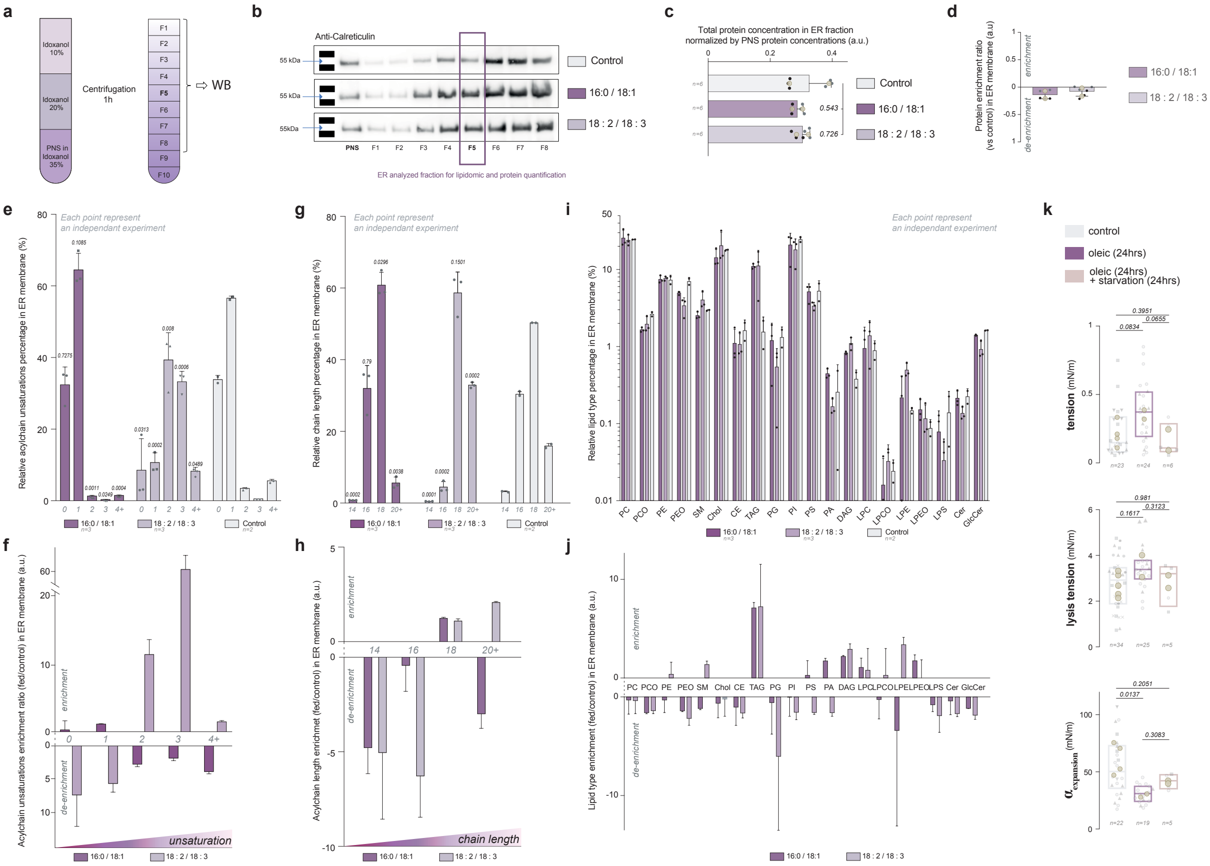

Supplementary Figure 4, related to Figure 2

a. Schematic illustration of the fractionation protocol using gradient centrifugation. Western blot analysis was performed on fractions F1 to F8.

b. Western blot analysis of the membrane fraction (F1 to F8) and post-nuclear supernatant (PNS) fraction, using an anti-calreticulin antibody to reveal the ER. Fraction F5 was selected for protein and lipid analysis under three different conditions: control COS7 cells (referred to as condition C), COS7 cells incubated with oleic:palmitic acid mixtures (200 µM:200 µM) for 24 hours, or COS7 cells incubated with linoleic:linolenic acid mixtures (200 µM:200 µM) for 24 hours.

c. Plot showing the total protein concentration in the ER fraction, normalized by the PNS protein concentration, for the three conditions: control COS7 cells, COS7 cells incubated with oleic:palmitic acid mixtures, or COS7 cells incubated with linoleic:linolenic acid mixtures. No significant differences in total ER membrane protein were detected between the conditions. N=2 replicates, and n=3 repetitions of concentration determination with absorbance measurements for each condition. Nested One-Way ANOVA - Multiple comparison: Fisher LSD test. Individual P values are shown.

d. Plot showing the ratio of total protein in the ER of fed cells compared to control cells. No significant differences were found. N=2 replicates, and n=3 repetitions of concentration determination with absorbance measurements.

e. Plot showing the relative percentage of acyl chains (with 0, 1, 2, 3, 4+ unsaturation) found in ER fraction F5. N=3 for the lipid supplemental conditions, and N=2 for the control. Statistical analysis was performed by comparing both conditions A and B with C. Unpaired t-test with two-tailed P-value. P-values are shown.

f. Plot showing the ratio of acyl chain enrichment in the ER membrane of the lipid supplemental conditions compared to control. N=3 for the lipid supplemental conditions, and N=2 for the control. This plot shows variations in the relative abundance of acyl chains with 0, 1, 2, 3, or more unsaturation in the ER membrane extracted from cells fed with different lipid mixtures (oleic/palmitic: de-enrichment for 2, 3, and 4+ unsaturations; linoleic:linolenic: de-enrichment for 0 and 1 unsaturation; enrichment for 2, 3, 4+ unsaturation).

g. Plot showing the relative percentage of chain length (ranging from 14C, 16C, 18C, and 20C+ carbons) found in ER fraction F5. N=3 for the lipid supplemental conditions, and N=2 for the control. Statistical analysis was performed by comparing both the lipid supplemental conditions vs. the control.

h. Plot showing the ratio of chain length enrichment in the ER membrane of the lipid supplemental conditions compared to the control. N=3 for the lipid supplemental conditions, and N=2 for the control. This plot shows variations in the relative abundance of acyl chains with 14C, 16C, or 20C+ carbons (oleic/palmitic: de-enrichment for 14C and 20+C carbons; linoleic:linolenic: de-enrichment for 14C+ and 16C+ chains; enrichment for 20+ chains). Unpaired t-test with two-tailed P-value. P-values are shown.

i. Plot showing the relative percentage of lipid species abundance found in ER fraction F5. N=3 for the lipid supplemental conditions, and N=2 for the control condition.

j. Plot showing the lipid species abundance enrichment ratio in the ER membrane of the lipid supplemental conditions compared to the control condition. N=3 for the lipid supplemental conditions, and N=2 for the control condition. No significant differences were observed in the polar head groups of lipids found in the ER membrane between

the different conditions.

k-top: Plot showing the lysis tensions of GERVs derived from cells under different metabolic conditions (control; 24-hour feeding with oleic acid; 24-hour feeding with oleic acid followed by 24-hour starvation). For conditions from left to right, the number of independent experiments is N=4; N=2; N=2; Each experimental data point corresponds to a GOV from a different cell, and the mean for each replicate is shown. Nested One-Way ANOVA - Multiple comparisons: Fisher LSD test. Individual P values are shown.

k-middle: Plot showing the initial membrane tensions of GERVs derived from cells under different metabolic conditions (control; 24-hour feeding with oleic acid; 24-hour feeding with oleic acid followed by 24-hour starvation). For conditions from left to right, the number of independent experiments is N=5; N=2; N=2; Each experimental data point corresponds to a GOV from a different cell, and the mean for each replicate is shown. Nested One-Way ANOVA - Multiple comparisons: Fisher LSD test. Individual P values are shown.

k-bottom: Plot showing the apparent area expansion modulus (α_expansion) of GERVs derived from cells under different metabolic conditions (control; 24-hour feeding with oleic acid; 24-hour feeding with oleic acid followed by 24-hour starvation). For conditions from left to right, the number of independent experiments is N=3; N=2; N=2; Each experimental data point corresponds to a GOV from a different cell and the mean for each replicate is shown. Nested One-Way ANOVA - Multiple comparison: Fisher LSD test. Individual P values are shown.

(Fig c, e, f: Median, 1st and 3rd quartiles, data point values. See Supplementary Data Table 6 for statistical analysis and data sets).

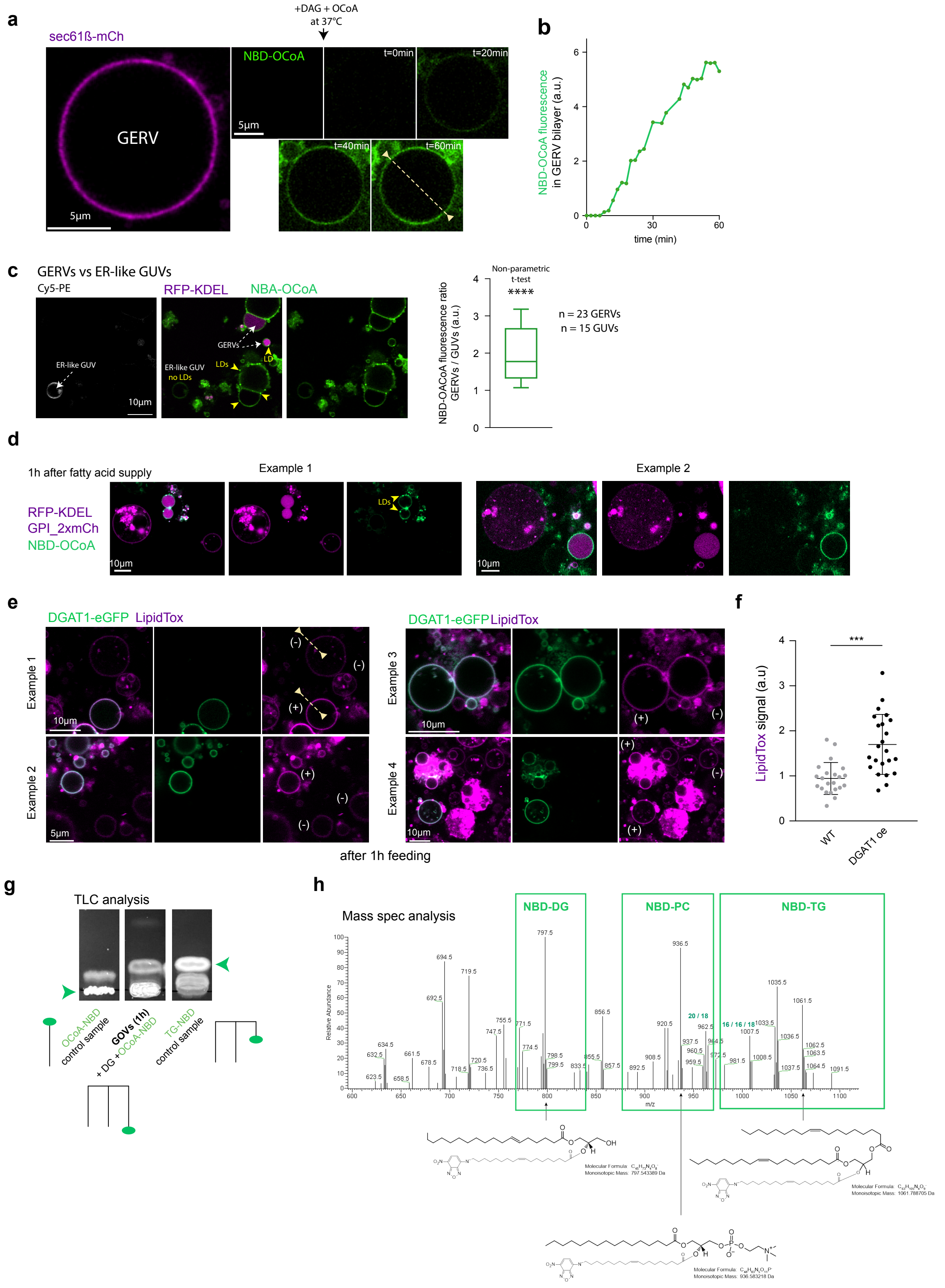

Supplementary Figure 5, related to Figure 3

a. Confocal microscopy image of a Sec61ß-based GERV. The GERV was treated with OCoA (Oleoyl-Coenzyme A) supplemented with 1% NBD-OCoA, and DAG (Diacylglycerol) at 37°C. The incorporation of fluorescent NBD-OCoA into triglycerides in the GERV membrane can be observed over time.

b. Plot showing the fluorescence signal of NBD-OCoA in the GERV membrane during the feeding experiment. The fluorescence intensity increases with time.

c. Left. ER-like GUVs (Giant Unilamellar Vesicle) and GERVs are brought into contact before being supplemented with NBD-OA-CoA. Lipid droplets (LDs) can form on the flat membrane of the GERV, while no LDs appear on the ER-like GUVs. Right. Comparison of the fluorescence signal of NBD-OCoA in GERVs versus ER-like GUVs. There is a higher NBD signal in GERVs likely due to the active synthesis of NBD-tagged triglycerides. The plot represents the data using a box plot, showing the median, 1st and 3rd quartiles, maximum, and minimum values. Student t-test with two-tailed P-value. P-value is < 0.0001.

d. Confocal microscopy image of KDEL-based GERV and GPI-based GPMV after 1 hour of treatment with OCoA (+ NBD-OCoA) and DAG. A green fluorescence signal is visible on the GERV membrane but not on the GPMV-like vesicle membrane.

e. DGAT1-based GERVs were treated with OCoA (+ NBD-OCoA) and DAG for 30 minutes. LipidTox, a fluorescent dye reporting membrane hydrophobicity, was added before imaging. Confocal microscopy images show DGAT1-based GERVs and GOVs, with fluorescence signal from LipidTox. Four examples are shown, with (+) and (-) indicating GOVs positive and negative for DGAT1-eGFP, respectively.

f. Plot showing the fluorescence signal of LipidTox in vesicles positive or negative for DGAT1-eGFP. DGAT1-based GERVs exhibit a higher LipidTox signal compared to other GOVs, indicating the presence of triglycerides in their membrane.

g. Thin-layer chromatography results of GERVs fed with NBD-OCoA and DAG at 37°C for 1 hour. Lipid extracts were then deposited on a silica plate and subjected to migration using a solvent system containing hexane, diethyl ether, and sulfuric acid. Commercial samples of NBD-OCoA and TAG36C-NBD were added to visualize the migration of TAG48C-NBD synthesized from GERVs.

h. Mass spectrometry analysis of lipid extracts obtained from GERV samples treated with NBD-OCoA and DAG at 37°C for 1 hour. The plot shows the relative abundance of lipid species as a function of their m/z (mass-to-charge) value. This plot allows the identification of TAG-NBD, PC-NBD, and DG-NBD species produced from GERVs. The green rectangles indicate the areas corresponding to these three different species.

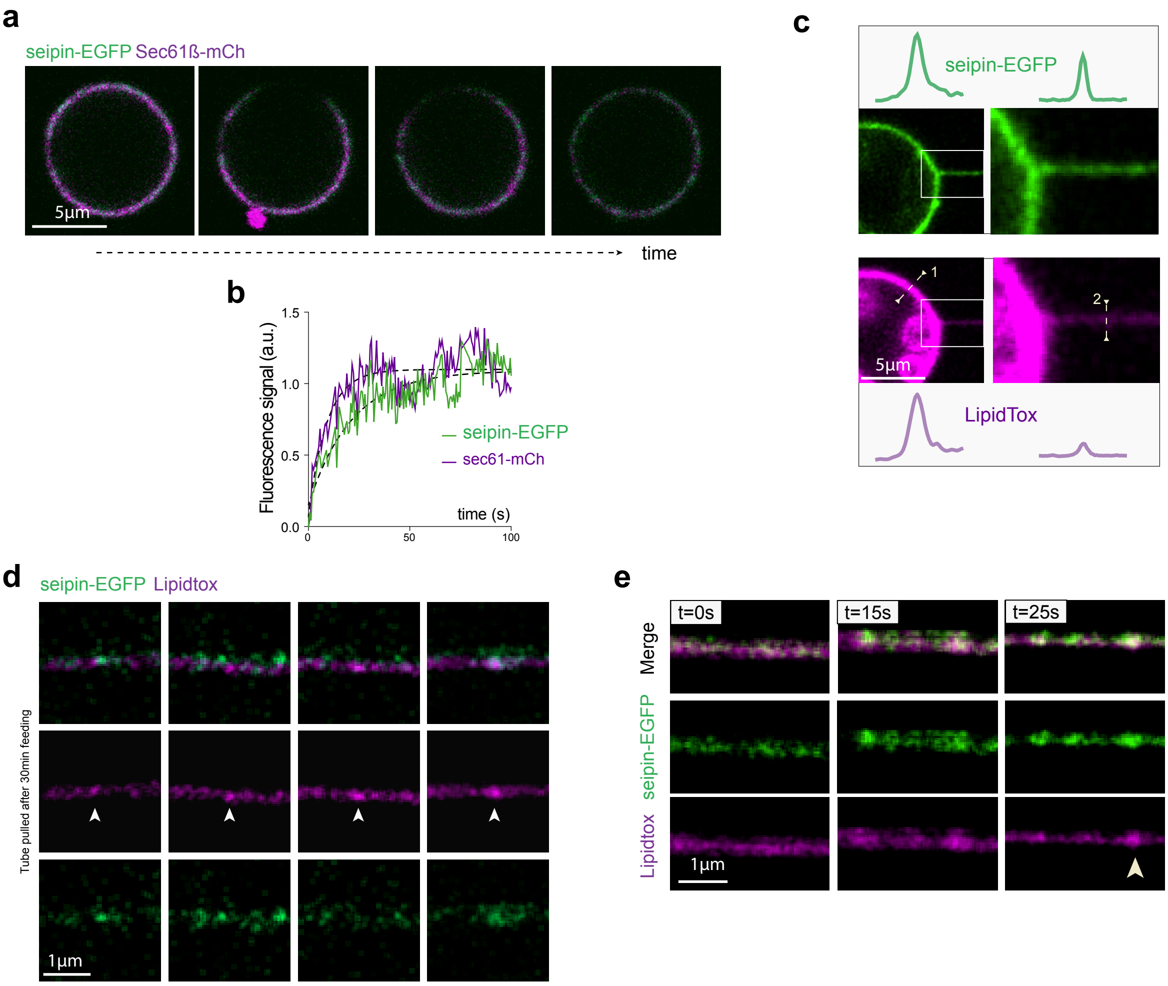

Supplementary Figure 6, related to Figure 4

a. Confocal microscopy image of a time course during a photo-bleaching experiment on a (Sec61ß & seipin)-based GERV. Both Sec61ß-mCh and seipin-EGFP were bleached.

b. Plot showing the mean fluorescence signal in the bleached region of the GERV over time. The signal is normalized by the fluorescence signal in the entire GERV. The fluorescence signals of Sec61ß-mCh and seipin-EGFP gradually recover over time, indicating the diffusivity of these proteins within the GERV membrane.

c. Confocal microscopy snapshot of a nanotube extracted from a seipin/Sec61ß-based GERV. Fluorescence profiles are drawn perpendicular to the membrane in both the flat region (1) and the nanotube (2).

d. Time course showing the formation of a lipid droplet on a seipin-based GERV tubule, visualized using LipidTox Deep Red. The fluorescence signal in LipidTox increases over time, indicating the accumulation of lipids in the droplet.

e. Time course showing the formation of a lipid droplet on a seipin-based GERV tubule, visualized using LipidTox Deep Red. The fluorescence signal in LipidTox increases over time at some seipin clusters (indicated by an arrow) but not all of them.
